# Papain-Based Solubilization of Decellularized Extracellular Matrix for the Preparation of Bioactive, Thermosensitive Pregels

**DOI:** 10.1021/acs.biomac.3c00602

**Published:** 2023-11-27

**Authors:** Ahed Almalla, Laura Elomaa, Leïla Bechtella, Assal Daneshgar, Prabhu Yavvari, Zeinab Mahfouz, Peter Tang, Beate Koksch, Igor Sauer, Kevin Pagel, Karl Herbert Hillebrandt, Marie Weinhart

**Affiliations:** † Institute of Chemistry and Biochemistry, Freie Universität Berlin, 14195 Berlin, Germany; ‡ Experimental Surgery, Department of Surgery, CCM|CVK, Charité − Universitätsmedizin Berlin, Augustenburger Platz 1, 13353 Berlin, Germany; § Fritz Haber Institute of the Max Planck Society, Faradayweg 4-6, 14195 Berlin, Germany; ∥ Berlin Institute of Health at Charité − Universitätsmedizin Berlin, BIH Biomedical Innovation Academy, BIH Charité, Clinician Scientist Program, Charitéplatz 1, 10117 Berlin, Germany; ⊥ Institute of Physical Chemistry and Electrochemistry, Leibniz Universität Hannover, 30167 Hannover, Germany

## Abstract

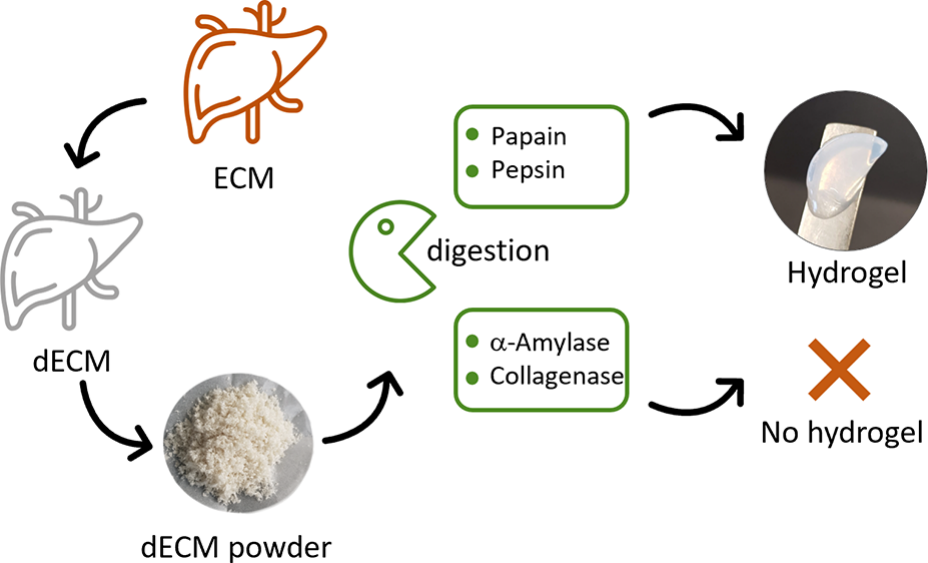

Solubilized, gel-forming decellularized extracellular matrix (dECM) is used in a wide range of basic and translational research and due to its inherent bioactivity can promote structural and functional tissue remodeling. The animal-derived protease pepsin has become the standard proteolytic enzyme for the solubilization of almost all types of collagen-based dECM. In this study, pepsin was compared with papain, α-amylase, and collagenase for their potential to solubilize porcine liver dECM. Maximum preservation of bioactive components and native dECM properties was used as a decisive criterion for further application of the enzymes, with emphasis on minimal destruction of the protein structure and maintained capacity for physical thermogelation at neutral pH. The solubilized dECM digests, and/or their physically gelled hydrogels were characterized for their rheological properties, gelation kinetics, GAG content, proteomic composition, and growth factor profile. This study highlights papain as a plant-derived enzyme that can serve as a cost-effective alternative to animal-derived pepsin for the efficient solubilization of dECM. The resulting homogeneous papain-digested dECM preserved its thermally triggered gelation properties similar to pepsin digests, and the corresponding dECM hydrogels demonstrated their enhanced bioadhesiveness in single-cell force spectroscopy experiments with fibroblasts. The viability and proliferation of human HepaRG cells on dECM gels were similar to those on pure rat tail collagen type I gels. Papain is not only highly effective and economically attractive for dECM solubilization but also particularly interesting when digesting human-tissue-derived dECM for regenerative applications, where animal-derived materials are to be avoided.

## 1 Introduction

Biologic scaffolds composed of an extracellular matrix (ECM) can be created by removing cellular components of tissues or organs via various approaches, including physical, chemical, and enzymatic methods. The process itself, which ideally leaves behind an intact and highly preserved meshwork of ECM components with tissue-specific composition and architecture, is referred to as decellularization.^1^ Decellularized extracellular matrix (dECM) has gradually become the gold standard among scaffolds for tissue engineering because, ideally, the immunogenic cellular components are completely removed, while the composition, architecture, and topology of the native cell environment are preserved.^2^ In native tissues, cells generate and maintain the ECM, which is mainly composed of a variety of structural and regulatory proteins, including glycosaminoglycans (GAG), proteoglycans, cytokines, and growth factors, that play an important role in multiple essential cellular processes, such as migration, proliferation, and differentiation.^3^ In pursuit of the ultimate scaffold for tissue engineering, within the last few decades, various materials have been proposed. Many of them are based on individual ECM components, such as collagen, laminin, hyaluronic acid, and their combinations, or a gelatinous, undefined basement membrane protein mixture derived from mouse tumor cells commercialized as Matrigel.^4^ Although these materials can improve cellular viability and function compared with inert materials, they demonstrate incomplete bioactivity because they lack the complexity of native ECM.

Tissue-derived dECM overcomes these limitations with its preserved matrix composition and protein ultrastructure. It can be used in its native insoluble form, which features tissue architecture, including the vascular tree, or as a solubilized material that can form injectable hydrogels. Solid, insoluble dECM scaffolds, including dECM sheets/membranes^5^ and whole organs, can be categorized based on their application.^6^ A major limitation of these scaffolds is their batch-to-batch variability since tissue sheets cannot be mixed or blended, which is otherwise a common approach to cope with biological variability. Additionally, the fixed geometric shape and mechanical properties of the dECM scaffolds limit their use in tissue engineering applications, where flexibility in shape and design with tunable stiffness are required. In contrast, soluble or flowing dECM materials can be categorized based on the method of their reconstitution or application, which includes dECM suspensions/slurries, injectable hydrogels, 2D and 3D hydrogels or coatings, bioinks, and -resins for 3D bioprinting and combinatorial cross-linked hybrid patches composed of solubilized dECM and synthetic biomaterials.^7^ Since soluble dECM materials are homogenized, batch-to-batch variability can be reduced by organ or tissue pooling. This enables the creation of dECM-based products with finely tuned reproducible properties for a wide range of applications.

For in vitro applications, dECM-derived hydrogels are the most commonly used type of processed dECM, particularly since the rise of 3D (bio)printing. Since 1998, when the first enzymatic ECM solubilization and subsequent gel formation were established,^8^ its application range has been expanded from basic to translational research.^9^ The formation of dECM hydrogels starts with transforming naturally cross-linked dECM into soluble monomeric or multimeric fragments while preserving its tissue-specific biochemical properties. With fibrous collagen being the major cross-linked component of dECM, it is typically solubilized in acidic conditions via enzymatic digestion with pepsin, as first reported for decellularized small intestinal submucosa by Voytik-Harbin et al.^8^ Pepsin is an animal-derived acidic proteolytic enzyme first extracted from the porcine stomach and has been used since the 1960s to solubilize acid-insoluble collagen.^10^ Similar to collagen, rapid self-assembly of the dissolved dECM back into a physically cross-linked hydrogel network can be induced by first adjusting its pH and salt concentration to physiological conditions, at the same time deactivating the digesting pepsin and subsequent incubation at 37 °C. The digestion time needs to be optimized for each tissue type/source and application; times of 24–96 h have been reported.^11^ However, despite the wide use of porcine pepsin in dECM digestion, its animal origin may raise regulatory or religious issues for potential future medical products. Hence, using nonanimal-derived, low-cost alternatives might be advantageous when digesting human dECM or aiming at upscaling processes. As a replacement for the protease pepsin, the glycolytic enzyme α-amylase has recently been reported in a milder digestion protocol for dECM, which cleaves carbohydrate groups from the collagen’s telopeptide region, thereby increasing its acid solubility.^12^ α-Amylase can be extracted from human or animal pancreatic juice and saliva or bacteria such as *Bacillus subtilis*. In 2013, Yu et al. applied α-amylase to successfully solubilize human-derived dECM in slightly acidic conditions (pH 5.4) in a two-step process.^12^ Although dECM-derived hydrogels produced from enzymatic digestion with α-amylase or pepsin both possess high viscosity, Kornmuller et al. reported that microcarriers prepared from α-amylase digests had increased mechanical properties compared to pepsin digests.^13^ While the influence of digestive pepsin and α-amylase on the resulting physical and biochemical properties of dECM pregels and hydrogels has been studied, other enzymes have not been comparatively evaluated for their solubilization efficiency and the resulting dECM properties.^11,12,14,15^ Thus, we further included the bacterial-derived enzyme, collagenase, which is widely used for the digestion of ECM when isolating cells and nuclear material from tissues, and plant-derived papain due to its common use when preparing dECM-samples for glycosaminoglycan (GAG) and deoxyribonucleic acid (DNA) quantification However, to our knowledge, both enzymes appear to be confined to these specific areas, and there are no reports on their utilization in generating dECM hydrogels.^16^

Because hydrogels derived from dECM are promising materials for tissue engineering and additive manufacturing, controlling the resulting physical, mechanical, and biochemical properties after enzymatic solubilization and gelation is important. Herein, we report a comprehensive study on the preparation of porcine liver dECM-derived hydrogels using four different enzymes from various sources, focusing on cost-effectiveness, solubilization efficiency, and maximum preservation of the original dECM properties and subsequent gel formation. Animal-derived pepsin, bacterial-derived α-amylase, and collagenase as well as plant-derived papain were tested and compared in terms of their solubilization efficiency and the chemical, physical, and biological characteristics of the digests. In the further course of our study, decisive criteria were applied to exclude enzymes from further evaluation. Therefore, the focus of the comparative study was finally limited to the two best-performing enzymes, papain, and pepsin.

## 2 Materials and Methods

### 2.1 Materials, Reagents, and Buffers

Pepsin from porcine *gastric mucosa* (#P7012), collagenase type II from *Clostridium histolyticum* (#C2-22-BIOC), papain from *Carica papaya* (#1.07144), α-amylase type II-A from *Bacillus subtilis* (#A6380), and proteinase K from *Tritirachium album* (#p6556) were purchased from Sigma-Aldrich (St. Louis, MO, United States) or MB biomaterials (Neustadt-Glewe, Germany) and used without further processing. Sodium dodecyl sulfate (SDS), Triton X-100, 1,9-dimethyl-methylene blue (DMMB), glycine, chondroitin sulfate, chloramine-T, *p*-dimethyl amino-benzaldehyde (DMAB), *trans*-4-hydroxy-l-proline, peracetic acid (38–40%), β-mercaptoethanol, bromophenol blue, Coomassie brilliant blue, dithiothreitol, 2-chloroacetamide, hydrocortisone-21-hemisuccinate (#H2270), insulin 5 (#I9278), penicillin/streptomycin (p/s) and glutamine, phosphate-buffered saline PBS (−/−) tablets (#4417), 2-(*N*-morpholino)ethanesulfonic acid (MES), ethylenediamine tetraacetic acid disodium salt (EDTA-Na_2_), l-cysteine (#168149), Tris-Base (#T1503), Tris HCl (#108319), thiourea (#T8656), urea (#U5128), dithiothreitol (DTT, # 3483-12-3), and ammonium bicarbonate (#A6141) were purchased from Sigma-Aldrich (St. Louis, MO, United States) and used as received. Protease and phosphatase inhibitors (cOmplete and PhosSTOP) were purchased from Roche Diagnostics GmbH (Mannheim, Germany). 3-[(3-cholamidopropyl) dimethylammonio]-1-propanesulfonate (CHAPS, #17038.03) was purchased from SERVA (Heidelberg, Germany). For bottom-up proteomics, lyophilized trypsin (no. V5111) was purchased from Promega Corporation (Wisconsin, USA). ZipTip analytical sample preparation pipet tips with 0.6 μL C18 resin, 10 kDa Ultracel Amicon Ultra 0.5 centrifugal filter units, potassium chloride (#104936), glycerol (#104094), and benzonase (purity >90%, #70746-3) were purchased from Merck Chemicals GmbH (Darmstadt, Germany). Precision Plus Protein Unstained Standards was purchased from Bio-Rad (Feldkirchen, Germany). Pierce RIPA buffer (#89900), Halt protease, phosphatase inhibitor cocktail (#78440), William’s E cell culture medium, PrestoBlue reagent, Pierce LAL chromogenic endotoxin quantitation kit, fluorescein diacetate (FDA, #F7378), propidium iodide (PI, #P4170), and Hoechst (#33342) were purchased from Thermo Fisher Scientific (Waltham, MA, United States). Collagen type I from rat tail was purchased from Bio-Techne (R&D Systems, Minnesota, USA). Human HepaRG 101 cells were purchased from Biopredic International (Saint-Grégoire, France). Fetal bovine serum (FBS) was obtained from PAN Biotech (Wimborne, UK). Cell-TAK was obtained from Corning (New York, NY, United States), and PBS containing bivalent calcium and magnesium ions was obtained from Gibco (Darmstadt, Germany). Fine-meshed nylon filters were purchased from Oriental Riverkit (Wuhan, China).

Ehrlich’s reagent was freshly prepared as 0.1 M DMAB in a 68:32% v/v mixture of *n*-propanol and 70% perchloric acid. Chloramine-T working solution was prepared as 80:10:10% v/v of citric acetate buffer, *n*-propanol, and H_2_0, respectively, and stored at RT. Citric acetate buffer containing 5% citric acid, 7.24% sodium acetate, and 1.2% glacial acetic acid in H_2_0 was adjusted to pH 6.0 with NaOH. The DMMB dye solution was prepared by dissolving the dye (16 mg), glycine (3.04 g), and NaCl (1.6 g) in 0.1 M HCl (95 mL), and the volume was adjusted with distilled water to 1 L, resulting in pH 3. The lysis buffer (pH 8) for double-stranded DNA (dsDNA) extraction contained 50 mM Tris–HCl, 25 mM EDTA, 400 mM NaCl, and 10 μg of proteinase K. CHAPS-Tris buffer was prepared from 112 mM CHAPS, 50 mM Tris-Base, and 50 mM potassium chloride at pH 7.5 supplemented with cOmplete and PhosSTOP inhibitors cocktail. SDS protein lysis buffer (pH 6.8) was prepared from 50 mM Tris–HCl, 2% SDS, 10% glycerol, 1% β-mercaptoethanol, 12.5 mM EDTA, and 0.02% bromophenol blue.

### 2.2 Decellularization of Porcine Liver

Three porcine livers were freshly harvested after an experiment approved by the State Office of Health and Local Affairs (LAGeSo, Berlin, Germany) and stored at −20 °C until further processing. The thawed porcine livers were sectioned into 0.5–2 mm thick slices using a razor blade and scissors. Representative native tissue pieces were deferred for the quantitative analysis of dsDNA, sulfated GAG (sGAG), and collagen via the hydroxyproline content before decellularization. The pooled tissue slices were transferred into distilled H_2_O or PBS (−/−) at 4 °C in 2L beakers equipped with a magnetic stir bar with several changes of the suspending media within 24 h until the blood was completely removed and tissue slices appeared pale-yellow. Generally, the suspending medium throughout the decellularization process was exchanged by filtration of the sliced tissues through fine-meshed nylon filters and resuspension of the recovered solids in fresh medium. Next, PBS (−/−) was replaced with 1% SDS solution, agitated for 2 h, and further replaced with 1% Triton X-100 for another 24 h. The filtered tissue slices were washed gently for 30 min with distilled water before the tissue was defatted for 30 min in pure ethanol. The tissue slices were quickly sterilized in a suspension with 0.1% aqueous peracetic acid for 20 min, after which the peracetic acid was continuously replaced with PBS (−/−) and then distilled water for at least 3 days [minimum 2–3 fresh PBS (−/−) or distilled water changes a day]. After filtration, the filtrate was snap-frozen with liquid nitrogen before lyophilization, and the obtained decellularized, lyophilized tissue slices were ground with the help of a mortar/pestle or an electrical coffee mill after shock-freezing the samples with liquid nitrogen to obtain fine dECM powders. The powder was stored at −20 °C until it was used for experiments.

### 2.3 Enzymatic Digestion of Porcine Liver dECM

**Figure 1:**
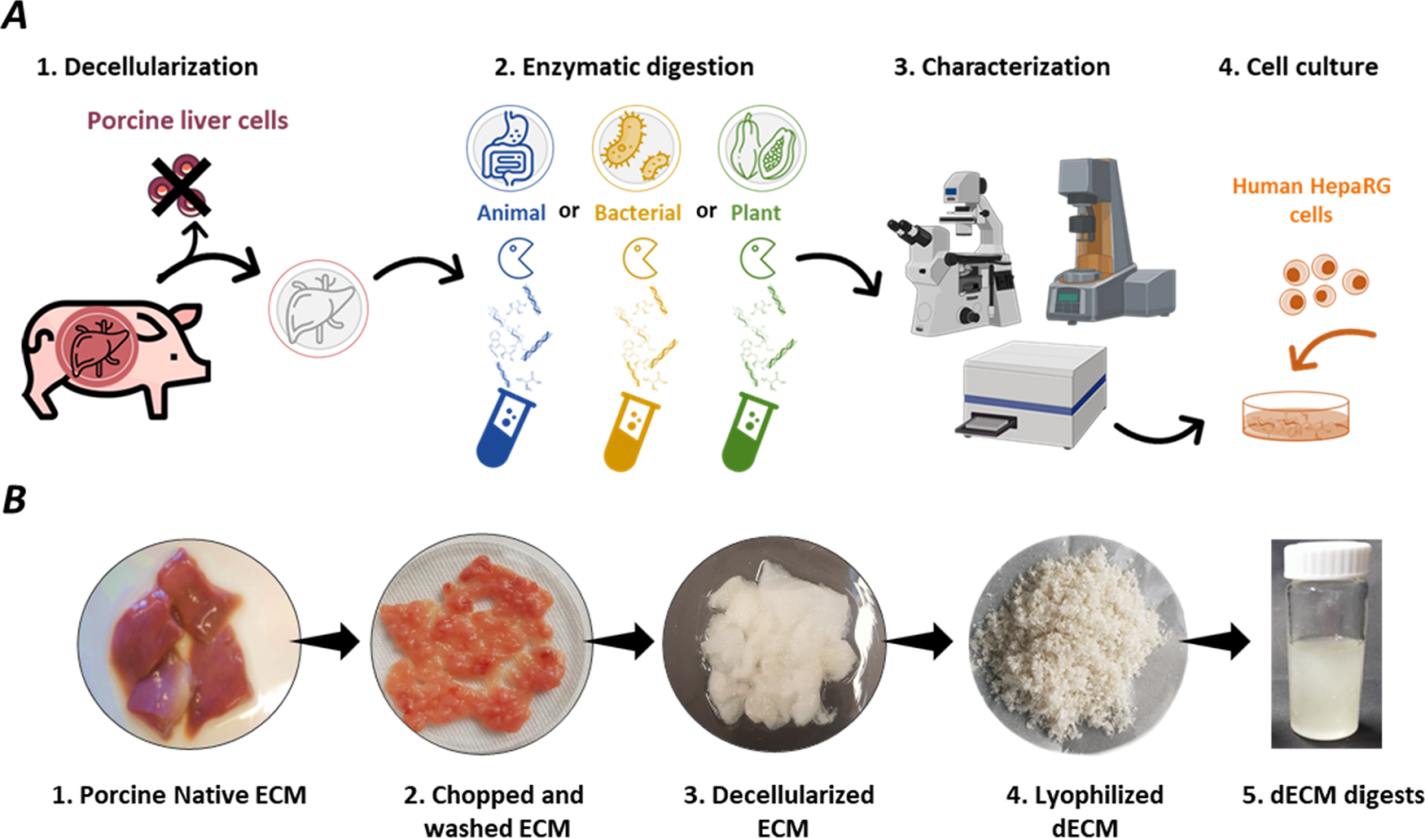
(A) Schematic experimental approach including the comparative enzymatic digestion of porcine liver dECM with different enzymes, the characterization of the respective digests, and their final performance after gelation in cell culture with human HepaRG cells. (B) Representative photographs of the preparation of homogeneous dECM digests, which are liquid pregels at 4 °C under acidic conditions.

Selected enzymes from animal, bacterial, and plant origins were used for liver dECM solubilization and hydrogel formation, as outlined in Figure 1.

Enzyme solutions were prepared with an adequate enzyme concentration considering the respective supplier-stated activity and recommendations and were further adjusted according to the results from initial screening experiments with a visual assessment of their solubilizing potential (representative pictures are shown in Figure S1). Specific pH-adjusted buffers or solutions were used according to the manufacturer’s instructions, with additional activators added as needed and considering already published protocols (Table 1).^11,15,17^ The temperature during the digestion process was set within the optimal activity range of the respective enzyme, avoiding temperatures above 37 °C to minimize the possibility of unnecessary protein denaturation. In general, room temperature (∼25 °C, RT) was preferred for the highly active enzymes to allow for convenient digestion procedures.

**Table 1:** Description of Enzymatic Specificities and Conditions Used in This Study

enzyme	activity^a^ (units/mg protein)	aqueous buffer or solution	pH	*T* (°C)	enzyme concentration (mg/mL)^b^	digestion steps
papain	30,000 USP	20 mM EDTA, 5 mM l-cysteine	5.5 ^17^	25	0.5	1
pepsin	≥2500	0.01 M HCl	2.0 ^11^	25	1	1
α-amylase	≥1500	0.22 M NaH_2_PO_4_	5.0 ^15^	25 or 37	1.5	2
collagenase	≥200	50 mM CaCl_2_, 50 mM MES	7.0	37	0.1	1

a As stated by the supplier.

b For a concentration of 10 mg/mL dry dECM in digestion media.

The enzyme solutions were mixed with the lyophilized dECM powder (10 mg/mL), homogenized using a homogenizer (T 10 basic Ultra-Turrax, IKA-Werke GmbH & Co. KG), and constantly agitated for 24 h at either 25 or 37 °C as specified in Table 1. After 24 h of digestion with α-amylase, the enzyme was inactivated by shifting the pH to acidic conditions (pH = 2) on ice for 1 h.^18,19^ Subsequently, the dECM-Amylase digest was allowed to stir for another 24 h at pH 2 as a second nonenzymatic step of protein solubilization. For one-step digestions with papain, pepsin, and collagenase, the 48 h digests were inactivated on ice for 1 h by changing the pH to 9.5–10 for papain, pH 9 for pepsin, and pH 2 for collagenase.^17−20^ All samples were periodically homogenized using an IKA Ultra-Turrax mixer during the digestion. Finally, all dECM digests were centrifuged gently at 140*g* for 5 min to remove any undigested particles, if present. Supernatants were purified via dialysis (SpectraPor with molecular weight cutoff of 1 kDa, Carl Roth GmbH + Co. KG (Karlsruhe)) first in distilled water and then in 0.01 M HCl for 48 h with 2–3 acid changes. The final dECM digests were snap-frozen with liquid nitrogen and lyophilized. Dry dECM foamlike digests were stored at −20 °C until further use.

### 2.4 Biochemical Characterization

#### 2.4.1 dsDNA Quantification

Isolation of genomic dsDNA from native ECM, dECM, and digested dECMs was carried out as follows. Dry samples (50 mg) were incubated in lysis buffer (820 μL) at 60 °C overnight under strong shaking and centrifuged at 10,000*g* for 10 min, and the supernatants were used without further purification. The dsDNA content was determined with a DNA quantitation kit (AccuBlue Broad Range, Biotium, USA) according to the manufacturer’s instructions. Briefly, 10 μL of each dsDNA standard or unknown sample was added to a well of a 96-well microplate, the fluorescent working solution (200 μL) was added and mixed, and the samples were incubated for 15 min in the dark at RT. A standard curve was prepared from the kit-derived set of dsDNA dilutions to calculate the dsDNA concentrations of the unknown samples. Fluorescence values were read with a microplate reader (Infinite M200 Pro, Tecan, Switzerland) with an excitation wavelength of 350 nm and an emission wavelength of 460 nm. Samples were run in triplicate, the DNA content was normalized to the dry weight of the sample, and data were presented as nanograms per milligram of dry tissues.

#### 2.4.2 Hydroxyproline Quantification

To quantify the hydroxyproline content, 10 mg of lyophilized native liver ECM, dECM, and dECM-digests was placed into an Eppendorf tube and suspended in 6 M HCl (100 μL) under continuous vortexing. Collagen was hydrolyzed overnight (∼16 h) at 115 °C, followed by centrifugation at 10,000*g* for 10 min. The supernatant was collected and used to chromogenically quantify the hydroxyproline content. Briefly, 5 μL of both standards and unknown samples were added in triplicate into the 96-well plate containing 50 μL of citric acetate buffer and then oxidized with freshly prepared 0.05 M chloramine-T (100 μL) in chloramine-T working solution for 30 min at RT. Finally, freshly prepared Ehrlich’s reagent (100 μL) was added to each well, mixed, and, after 5 min absorbance, measured at 550 nm as preincubation reading, and repeated after 25 min of incubation at 65 °C as postincubation reading with a microplate reader (Infinite M200 Pro, Tecan, Switzerland). By subtracting the pre- from the postincubation reading, the total hydroxyproline content was obtained with the help of a standard curve of *trans*-4-hydroxy-l-proline and normalized to dry tissue weight.

#### 2.4.3 sGAG Quantification

Extraction of sGAG from 10 mg lyophilized tissue samples of native liver ECM, dECM, and solubilized dECM was achieved after enzymatic treatment with 0.03 mg/mL papain in lysis solution (20 mM sodium phosphate buffer at pH 6.8, 1 mM EDTA-Na_2_ and 2 mM dithiothreitol) overnight at 60 °C under strong shaking. The samples were then centrifuged at 10,000*g* for 10 min, and the supernatants that contained the total soluble GAG content were analyzed using the 1,9-dimethylmethylene blue (DMMB) dye assay according to a previously published protocol.^21^ Briefly, 400 μL of sGAG standards (chondroitin sulfate) or unknown samples were mixed with freshly prepared and filtered DMMB dye solution (400 μL after diluting in DMMB buffer as 1:10), and the absorbance at 525 nm was measured immediately using UV–vis spectroscopy (Agilent Cary 8454, Agilent Technologies, USA) at RT. Samples were run in triplicate, compared to the chondroitin sulfate standard curve and normalized to the dry weight of the samples.

#### 2.4.4 Growth Factor Quantification

Growth factors were extracted from tissues by shaking 20 mg of lyophilized native liver ECM or dECM digests overnight at 4 °C in RIPA lysis buffer (1 mL) containing the Halt inhibitor cocktail (1×). After centrifugation, the supernatant was collected for Quantibody human growth factor multiplex ELISA array Q1 (RayBiotech, Norcross, GA, USA), which was used according to the manufacturer’s protocol without any modifications. The fluorescence signal was read at 532 nm using a GenePix 4300A microarray scanner (Molecular Devices, USA), and the concentration of the growth factors was quantified against a linear calibration curve of the respective growth factor.

#### 2.4.5 Proteomics Sample Preparation and Liquid Chromatography–Tandem Mass Spectrometry

To prepare the samples for shotgun proteomic analysis, a filter-aided sample preparation (FASP) protocol was used.^22^ To extract proteins, chilled CHAPS-Tris buffer (100 μL) was added to 10 mg of lyophilized dECM or dECM digests and sonicated. The samples were mixed with 2.5 μL of benzonase and 2 μL of 240 mM magnesium chloride containing Tris-buffer (pH 7.5, 50 mM Tris-Base, 50 mM potassium chloride, 20% glycerol) and incubated on ice. Next, 8 M urea (200 μL) in 0.1 M Tris–HCl (pH 8.5) and 2 M thiourea were added to all samples, followed by 10 min incubation at RT. All samples were processed according to a previously published protocol.^23^ In brief, protein extracts were transferred to moisten 10 kDa centrifugal filter units and centrifuged at 14,000 rpm for 15 min at 20 °C. From this point, all the following centrifugation steps were performed by applying the same settings. Next, 8 M urea in 0.1 M Tris–HCl (200 μL) plus 50 mM ammonium bicarbonate buffer (200 μL) were added to the samples, followed by centrifugation. Afterward, proteins were digested on a filter overnight by adding 40 μL of trypsin-containing ammonium bicarbonate buffer (20 μg trypsin resolved in 100 μL of 50 mM ammonium bicarbonate). To quench digestion, 50 mM ammonium bicarbonate buffer (50 μL) was pipetted to the samples. The digest was collected after centrifugation, desalted, and concentrated with a ZipTip pipet tip according to the manufacturer’s specifications.

Proteomic compositions were determined by employing liquid chromatography on a Dionex Ultimate 3000 Nano HPLC (Thermo Fischer Scientific, Waltham, MA 02451, USA) coupled to an Impact II ESI-Q-TOF mass spectrometer (Bruker Daltonics GmbH, Bremen 28359, Germany) as previously published.^23^ A 75 μm × 50 cm C18-silica packed column (Thermo Scientific #164939, Thermo Fisher Scientific, San Jose, USA) was used for peptide separation while the capillary column was kept at 60 °C and a mass range from 150 to 2200 *m*/*z* was scanned with a defined significance threshold of *p* < 0.05. Relative protein abundances were generated by label-free quantification. Mass spectra were analyzed by PEAKS Software (Bioinformatics Solutions Inc., ON N2L 3K8, Canada). A monoisotopic precursor search type was chosen, and the fragment ion mass tolerance was set to 0.05 Da. Oxidation and *N*-terminal acetylation were specified as variable modifications. The area under the curve (AUC) was utilized as a proxy for the relative protein abundance in each sample. Matrisome proteins were identified and categorized into six groups—collagens, ECM glycoproteins, proteoglycans, ECM regulators, ECM affiliated proteins, and secreted factors—using *MatrisomeDB*.^24,25^

### 2.5 Protein Structure and Molecular Weight Distribution in dECM Digests

#### 2.5.1 Fourier-Transformed Infrared and Circular Dichroism Spectroscopy

ATR-FTIR analysis was carried out on lyophilized and ground dECM powders (native and digested) using a Nicolet iS10 equipped with a smart diamond *ATR* accessory. 32 scans were conducted from 4000 to 600 cm^–1^ and averaged for each spectrum. The resolution and interval scanning were set at 4 and 2 cm^–1^, respectively. For circular dichroism (CD) spectroscopy, the digested dECM (1 mg) was dissolved in 7 mM phosphate buffer (1 mL, pH 7.4) and stirred overnight at 4 °C. Samples were centrifuged gently to remove any insoluble particles, and the supernatant was used for the CD measurements. The final protein concentration was adjusted to 0.1 mg/mL after concentration determination via UV–vis spectroscopy (Agilent Cary 8454, Agilent Technologies, USA) using a standard curve from rat tail collagen type I. The solution was added into a quartz cuvette (0.1 cm path length, Suprasil) and scanned with far-UV circular dichroism (CD) Jasco J-810 spectropolarimeter (Jasco GmbH) equipped with a HAAKE WKL recirculating chiller (Karlsruhe, Germany) and a Jasco PTC-423S Peltier temperature controller (Jasco GmbH) under a N_2_ atmosphere at 25 °C. The spectra were acquired from 250 to 190 at 0.2 nm intervals with a response time of 4 s and 3 averaged scans per sample. The buffer background was subtracted from the spectra. The mean residue ellipticity (deg · cm^2^/dmol) was calculated from millidegrees (m°) using an average molecular weight of an amino acid (120 g/mol) and the following equation: *m*°/*M*(10 · *L* · *C*), where *C* is the sample concentration in g/L, *M* is the average molecular weight of proteins (g/mol), and *L* is the path length of the cell (cm).

#### 2.5.2 SDS Polyacrylamide Gel Electrophoresis and Size Exclusion Chromatography

Soluble dECM-Papain and dECM-Pepsin digests (2 mg/mL, 10 μL) or commercial rat collagen type I (1 mg/mL, 10 μL) in 0.01 M HCl were mixed with SDS protein lysis buffer at a 1:1 ratio, heated to 90 °C for 10 min, and then cooled to RT. Polyacrylamide gels with a total monomer concentration of 7.5% were prepared following BIO-RAD hand-casting polyacrylamide gels guide, and the wells were loaded either with a protein sample (20 μL) or unstained natural protein standards (10 μL). Electrophoresis was conducted at 150 V for 45 min at RT using a Mini-protein tetra vertical electrophoresis cell (Bio-Rad, Feldkirchen, Germany). Protein bands were visualized by staining in Coomassie brilliant blue solution (1% (w/v) in 5% (v/v) methanol and 10% (v/v) acetic acid in H_2_O) for 20 min, followed by treatment in destaining solution (5% (v/v) methanol and 10% (v/v) acetic acid in H_2_O) overnight. The molecular weights of the resulting bands were approximated by the relative mobility of standard protein molecular weight markers. Following destaining, the gels were imaged using a ChemiDoc MP imaging system (Bio-Rad, Feldkirchen, Germany) with the Bio-Rad Image Lab software.

The proteins’ and protein fragments’ molecular weight distributions were further analyzed using size exclusion chromatography (SEC). Samples of dECM-Papain and dECM-Pepsin along with commercial collagen type I from rat tail as control at 2 mg/mL in 0.01 M HCl were first reduced and alkylated in one reaction step at 95 °C for 10 min using 1 volume of reducing buffer (20 mM DTT and 80 mM chloroacetamide in H_2_O) at a final sample concentration of 1 mg/mL. The SEC measurements were carried out at RT using a Dionex Nano Ultimate 3000 chromatographic system detecting the UV-absorbance at 214 nm on a Superose 6 Increase 3.2/300 column (Cytiva) with a bed volume of 2.4 mL at a flow rate of 0.1 mL/min in 50 mM phosphate buffer (150 mM NaCl, pH 7.4). Samples (2 μL, corresponding to 2 μg input) were injected for each run. Samples were run in triplicate, and the detected bands were fitted and integrated using OriginPro software (Version 2021b, OriginLab Corporation, Northampton, MA, USA).

### 2.6 Physical Properties of dECM Digests and Hydrogels

#### 2.6.1 Turbidimetric Gelation Kinetics and Fibrillogenesis

Gelation kinetics of the dECM-Pepsin and dECM-Papain digests were evaluated turbidimetrically using the microplate reader (Infinite M200 Pro, Tecan, Switzerland). Briefly, pregel solutions of dECM-Pepsin or dECM-Papain (10 mg/mL) or collagen type I from rat tail (2.5 mg/mL) as control were prepared in 0.01 M HCl and stored at 4 °C for 24 h. Next, all samples were neutralized using cold 1 M NaOH and cold 10× PBS (−/−) on ice to inhibit thermal cross-linking before the measurement. For each sample, 100 μL was loaded into a UV-compatible and transparent 96-well plate (UV-STAR Greiner, Bio-one). To prevent evaporation, we filled the other wells with distilled water. The plate reader was set to 37 °C, and the absorbance at 350 nm was measured every 1 min for a total of 120 min. The readings were scaled from 0 (initial absorbance) to 100% (maximum absorbance), or absorbance values were normalized from 0 to 1 according to the following equation: *NA* = (*A* – *A*_min_)/(*A*_max_ – *A*_min_), where *NA* indicates the normalized, *A* is the corresponding, *A*_min_ is the minimum, and *A*_max_ is the maximum absorbance. The gelation speed *S* was determined by calculating the growth portion slope of the normalized curves. The lag time (*t*_lag_) was derived from the intercept with the *x*-axis by extrapolating the linear part of the curve. The time required to reach 50 and 95% absorbance was denoted as *t*_50_ and *t*_95_, respectively.^26^ Measurements were repeated in triplicate with independent samples. The visual appearance and transparency of the gels were assessed photographically by placing them over a printed @-symbol at acidic and neutral conditions after a 2 h incubation time at 37 °C.

#### 2.6.2 Viscosity and Rheological Properties of Freshly Digested Pregels and Physically Cross-Linked Gels

The rheological and viscosity measurements were conducted using a rotational rheometer (Kinexus pro+, Malvern Panalytical) equipped with a 20 mm/1° upper cone plate geometry with a solvent trap and a Peltier system as a temperature controller for the plate. dECM-Papain (10 mg/mL), dECM-Pepsin, (10 mg/mL), and pure rat tail collagen (2.5 mg/mL) pregels were prepared in 0.01 M HCl and kept at 4 °C before the measurements. The steady-state shear viscosities of pregels were measured with a shear rate in the range of 0.01–100 s^–1^ at 25 °C. Pregels were placed on ice and cold 10x PBS (−/−), cold 0.1 M NaOH, and cold distilled water were added to the pregels for pH neutralization and final concentration adjustment. Before rheological measurements, the neutralized pregels were cast in a 48-well plate and incubated for 2 h at 37 °C to induce complete physical cross-linking. Afterward, hydrogels were transferred to a preheated rheometer plate, and any sample excess was trimmed using a spatula. The linear viscoelastic region of the samples was determined by an amplitude sweep test at 37 °C with a strain from 0.01 to 10% at a frequency of 1 Hz. The optimal strain was chosen to be 0.5% for the oscillation frequency test from 0.1 to 10 Hz at 37 °C. The measurements were repeated three times with independent samples in triplicate.

#### 2.6.3 Surface Stiffness, Morphology, and Single-Cell Force Spectroscopy via Atomic Force Microscopy

The dECM hydrogels were mechanically characterized via AFM stiffness mapping with a NanoWizard4 (JPK Instruments, Berlin, Germany) equipped with a PetriDishHeater instrument (JPK Instruments, Berlin, Germany) for in situ temperature-controlled measurements. The temperature was set to 37 °C throughout the experiments. To measure the surface Young’s modulus *E* of papain- and pepsin-digested dECM hydrogels (10 mg/mL), as well as rat collagen (2.5 mg/mL) samples, were prepared similarly to the above-mentioned rheology samples with a minimum diameter of 3 mm and a minimum thickness of 0.5 mm on Petri dishes and finally equilibrated in warm PBS (−/−). The sample thickness was set to be >0.5 mm to eliminate the effect of the underlying substrate for deep indentations. Measurements were done with triangular silicon nitride pyramidal tips attached to cantilevers with a nominal spring constant (k) of 0.03 N/m and a tip radius of 20 nm (MLCT-D, Bruker, Mannheim, Germany). Tip calibration and estimation of the spring constant were conducted by the thermal fluctuation method using the simple harmonic oscillator model on a bare Petri dish and in PBS (−/−) just prior to force spectroscopy experiments. For the AFM experiments, a *z*-length of 3 μm, a speed of 2 μm/s, and a set point of 0.5 nN were used to ensure a very gentle indentation. Controlled deformations were applied to the samples, and the compressive feedback forces were measured through cantilever deflection. Force–displacement (*F*–*z*) curves were produced by translating cantilever deflection (*d*) into force (*F*) by means of *F* = 1/4 k*d*, where k is the cantilever spring constant. The Young's modulus of the probed material was calculated by fitting the contact part of the measured approach force curves to a standard Sneddon model for a pyramidal indenter (tip) with a Poisson’s ratio of 0.5. The measurements were operated in force spectroscopy contact mode, wherein an array of 8 × 8 (64 points) of force–distance (*F–z*) curves was collected over the entire scan area of 10 × 10 μm. For each point on the grid, one or two sets of *F–z* curves were collected. By fitting the *F–z* curves to a contact mechanics Sneddon model, the compressive modulus was extracted. Morphological images of the samples were obtained with the same cantilever in quantitative imaging mode (QI) using a set point of 0.5 V and a *z*-length between 300 and 1000 nm. An area of 10 × 10 μm was chosen from the hydrogels for pixel-per-pixel scanning.

Single-cell force spectroscopy adhesion measurements were taken in the same setting as above but with 200 μm-long, tipless, v-shaped silicon nitride cantilevers with nominal spring constants of 0.06 N/m (NP-O, Bruker, Mannheim, Germany). The cantilever was calibrated similarly to the aforementioned MLCT cantilevers. Prior to the adhesion experiments, primary human fibroblasts (isolated from human dermal skin, passage numbers 6 to 8) were grown to about 80% confluency and detached from culture flasks by trypsin/EDTA and washed off with PBS (+/+) and then centrifuged (140 g for 3 min) and resuspended in PBS (+/+). Tissue culture Petri dishes with PDMS masks, as reported elsewhere,^27^ were washed and filled with PBS (+/+), after which they were warmed to 37 °C and allowed to equilibrate for 10 min before the addition of cell suspension. To attach a cell, the calibrated and adhesive cantilever (cantilever apex immersed for 20 min in 10% (v/v) cell-TAK in 0.1 M NaHCO_3_) was lowered onto a single cell at a constant velocity of 10 μm/s until an upward force of 5 nN was recorded. After 5 s, the cell-laden cantilever was raised by 5 μm and incubated for 10 min to ensure firm binding. For adhesion measurements, the single-cantilever-bound cell was moved over the dECM (10 mg/mL) or collagen (2.5 mg/mL) gel-coated wells made by PDMS masks and lowered onto the gel surface with a velocity of 2 μm/s. A 60 s lag between two successive approach–retraction cycles and a 30 s contact time interval were applied. The applied forces ranged between 0.25 and 0.40 nN with a maximum of 20–30 adhesion curves for each cell. Each hydrogel was tested with 6 to 7 cells, and experiments were repeated three times. Data were processed using JPK data processing software.

### 2.7 Ultrastructural Information Using Scanning Electron Microscopy

The internal fibrillar microarchitecture of the hydrogels was visualized by imaging the inner part of the lyophilized samples (ECM, dECM scaffolds, and gels). Gels of dECM-Papain, dECM-Pepsin, and collagen were prepared via thermally induced gelation for 2 h at physiological conditions of pH = 7.4 and 37 °C. The ECM, dECM scaffolds, and gels were blade-cut in two halves after lyophilization, and the inner surface was sputtered with a thin gold layer for subsequent imaging via SEM (Hitachi SU8030) at 15 kV.

### 2.8 Sterilization of Storable and Ready-to-Use Pregels

Both dECM-Papain and dECM-Pepsin pregels were sterilized directly after digestion in a dialysis bag (SpectraPor with molecular weight cutoff of 1 kDa, Carl Roth GmbH + Co. KG, Karlsruhe) via dialysis in either 1% of aqueous chloroform or 0.1% of aqueous peracetic acid for 1 h at RT. Dialysis was continued in sterile 0.01 M HCl for 4–5 days (1 or 2 acid changes daily under a sterile bench) at 4 °C. Final sterile, ready-to-use acidic pregels (∼6–8 mg/mL) were stored up to 1 year at either 4 or −20 °C without any detectable contamination or property alteration.

### 2.9 Cell Culture and Cell Studies

Hydrogels (pH 7.4 and 1× PBS (−/−)) from dECM-digests were prepared as described above at 10 mg/mL of either dECM-Papain or dECM-Pepsin and 2.5 mg/mL for collagen controls. For cell studies, 96-well microplates (ibidi GmbH, Germany) were coated directly with dECM pregels (70 μL per well) as a thin layer and left in an incubator for 60 min for complete physical cross-linking. Afterward, the second layer (30 μL) was added in the middle of the wells to avoid meniscus formation and was left in the incubator to cross-link for 2 h (hydrogel thickness was around 1 mm). Sterile hydrogel-coated surfaces can be stored in PBS (−/−) at 4 °C for up to 3 months without visual alterations. Before cell seeding, the hydrogel surfaces were rinsed twice with cell culture medium (William’s E medium supplemented with 10% FBS, 1% glutamine, 5 μg/mL insulin, 50 μM hydrocortisone-hemisuccinate, and 1% penicillin/streptomycin antibiotic solution), and the coated plates were prewarmed to 37 °C for 30 min. HepaRG cells (27 × 10^3^ cells/cm^2^, P16 to P18) suspended in medium (200 μL) were seeded on the gel-coated surfaces and grown under standard conditions (5% CO_2_, 37 °C, 95% humidity). The culture media were exchanged every 2–3 days. On days 1, 3, and 7 of cell culture, the viability of the cells was assessed by fluorescent live/dead staining. Therefore, the hydrogels were removed from the culture medium, rinsed with PBS (−/−), treated with live/dead staining solution (200 μL, 0.46 μM FDA/16 μM PI in PBS (−/−)) and nuclei counterstaining (5 μM Hoechst in media), and incubated for 10 min at RT. The stained cells were imaged using a 10x objective via confocal fluorescent microscopy (LSM800, Carl Zeiss, Jena, Germany). The metabolic activity of the cells was traced with a colorimetric PrestoBlue assay according to the manufacturer’s instructions. Briefly, a 1:10 dilution (200 μL) of the PrestoBlue reagent with cell culture medium) was added to adherent cells on the gel samples or a TCPS control and left to react for 1 h in an incubator (5% CO_2_, 37 °C, 95% humidity). For optical density (OD) measurements at 570 nm with a reference wavelength of 650 nm on a microplate reader (Infinite M200 Pro, Tecan, Switzerland), the supernatants (100 μL) were pipetted into fresh wells of a 96-well plate, measured, and compared to a standard calibration curve.

### 2.10 Statistical Analysis

The statistical significance of the gathered data was determined using a Kruskal–Wallis test followed by Dunn’s multiple comparisons post hoc test or one-way statistical analysis of variance (ANOVA) followed by a Tukey test using the OriginPro software (Version 2021b. OriginLab Corporation, Northampton, MA, USA). *p*-Values *p* < 0.05 were considered statistically significant and indicated by * or otherwise nonsignificant (n.s.).

## 3 Results

### 3.1 Decellularization and Solubilization of Liver ECM Using Different Enzymes

The identification of efficient solubilization protocols that maintain the capacity of solubilized dECM to form hydrogels under physiological conditions for cell culture applications involves four major steps: (1) tissue decellularization, (2) comparative enzymatic digestion of the resulting dECM with different enzymes to create pregels, (3) biophysical and biochemical characterization of the resulting nongelled and gelled dECM-digests, and (4) cytocompatibility testing of the resulting hydrogels (Figure 1A). Decellularization of frozen porcine livers in this study was initiated by thawing and mincing the native liver tissue into small pieces (1–2 cm). Freezing the organ or tissue not only provides convenient storage after harvesting but also aids the decellularization process through the subsequent freeze–thaw cycle. The small liver pieces were further ground using a scissor or a mechanical grinder into tiny pieces (1–5 mm) and subsequently subjected to heavy washing cycles with distilled H_2_O and PBS (−/−) to remove all residual blood. Further decellularization was facilitated by detergents. Iterative exposure to SDS and Triton-100× resulted in whitish, semitranslucent tissue segments (Figure 1B).^28^ To assist the subsequent enzymatic digestion, the resulting dECM was freeze-dried and cryo-ground to obtain more homogeneous small-sized particles for further processing with different enzymes. The characteristics of enzymes evaluated in this study, including their origin, relative cost, and obtained yields in liver dECM digestion, are summarized in Table 2.

**Table 2:** Enzyme Origin, Relative Average Cost per 10 g of Sales Unit, and Yield of Solubilized dECM Obtained after Digestion, Purification, and Lyophilization

enzyme	origin	cost^a^	yield ± SD % (*n* = 3)^b^
pepsin	animal-derived	high	86 ± 6
α-amylase	bacterial-derived	low-medium	10 ± 3
collagenase		low-medium	7 ± 2
papain	plant-derived	low-medium	79 ± 5

a Average enzyme’s cost range is derived from the CAS DataBase list, including a minimum of three suppliers (Table S1).

b Isolated yields ± standard deviation (SD) are calculated from soluble supernatants derived from 100 mg lyophilized native tissue after digestion, purification, and lyophilization according to the conditions stated in Table 1.

The liver dECM, as an intricate interlocking mesh of fibrillar and nonfibrillar proteins, is relatively inert against weak, low-activity enzymes based on our initial observations (not shown). Therefore, high-activity batches of the respective enzymes were generally employed (Table 1). The individual working solution and pH were adopted from the supplier with slight modifications, while the working temperature was set to RT for a convenient process and only increased to 37 °C for α-amylase and collagenase, for which the digestive capability and/or the intrinsic enzyme activity was low at RT. The pepsin concentration was chosen to be 1 mg/mL for a dECM concentration of 10 mg/mL according to our initial screening experiments with visual assessment (Figure S1) and in agreement with a standardized literature protocol.^11^ Due to the lack of standardized protocols for papain, α-amylase, and collagenase, minimum concentrations to digest the dECM effectively were chosen and adjusted (Figure S1). Higher enzyme concentrations were also tested but not applied to the final studies due to excessive digestion (collagenase) or no improved digestion (α-amylase).

The costs of an enzyme greatly depend on its stability and extractability, purity, and activity as well as the availability of the extraction source and sales unit. Due to complex extraction and purification processes, animal-based enzymes are often more expensive than plant- or microbial-derived enzymes (Table 2).^29,30^ Based on the CAS DataBase analyzing at least three suppliers, Table S1 presents a general overview of the enzyme price range irrespective of its activity. Further, prices per 10 g sales unit of the specific enzymes used in this study and their respective calculated costs for the digestion of 1 g liver dECM according to our working conditions (Table 1) are presented, identifying papain, α-amylase, and collagenase as highly cost-effective alternatives to pepsin.

Visual inspection revealed that pepsin, collagenase, and papain completely dissolved the liver dECM after 48 h of one-step digestion, whereas α-amylase resulted in only partial dissolution (Figure S1). Further extension of the digestion time up to 1 week, increasing the α-amylase concentration or digesting at a higher temperature (37 °C) did not increase the solubility of the residual dECM chunks (data are not shown). Therefore, an additional 24 h of acidic extraction step was introduced after 24 h of α-amylase digestion (Table 1). For further characterization, only the soluble supernatants were collected, purified, lyophilized, and considered. While both papain- and pepsin-digested dECM generated relatively high yields (≥79%) at RT, α-amylase and collagenase resulted in comparably low yields of ≤10% at 37 °C (Table 2). Since yields are solely based on the soluble fractions after purification (dialysis 1 kDa), low (or lost) yields can result from either undigested/insoluble dECM chunks that were removed by centrifugation or very low molecular weight fragments (<1 kDa) that are lost during the dialysis. Freeze-drying of the solubilized, enzyme-digested material resulted in a white powder that yielded viscous pregel solutions when dissolved in an acidic solution at 4 °C.

### 3.2 Biochemical and Structural Analysis of Enzyme-Digested dECM

**Figure 2:**
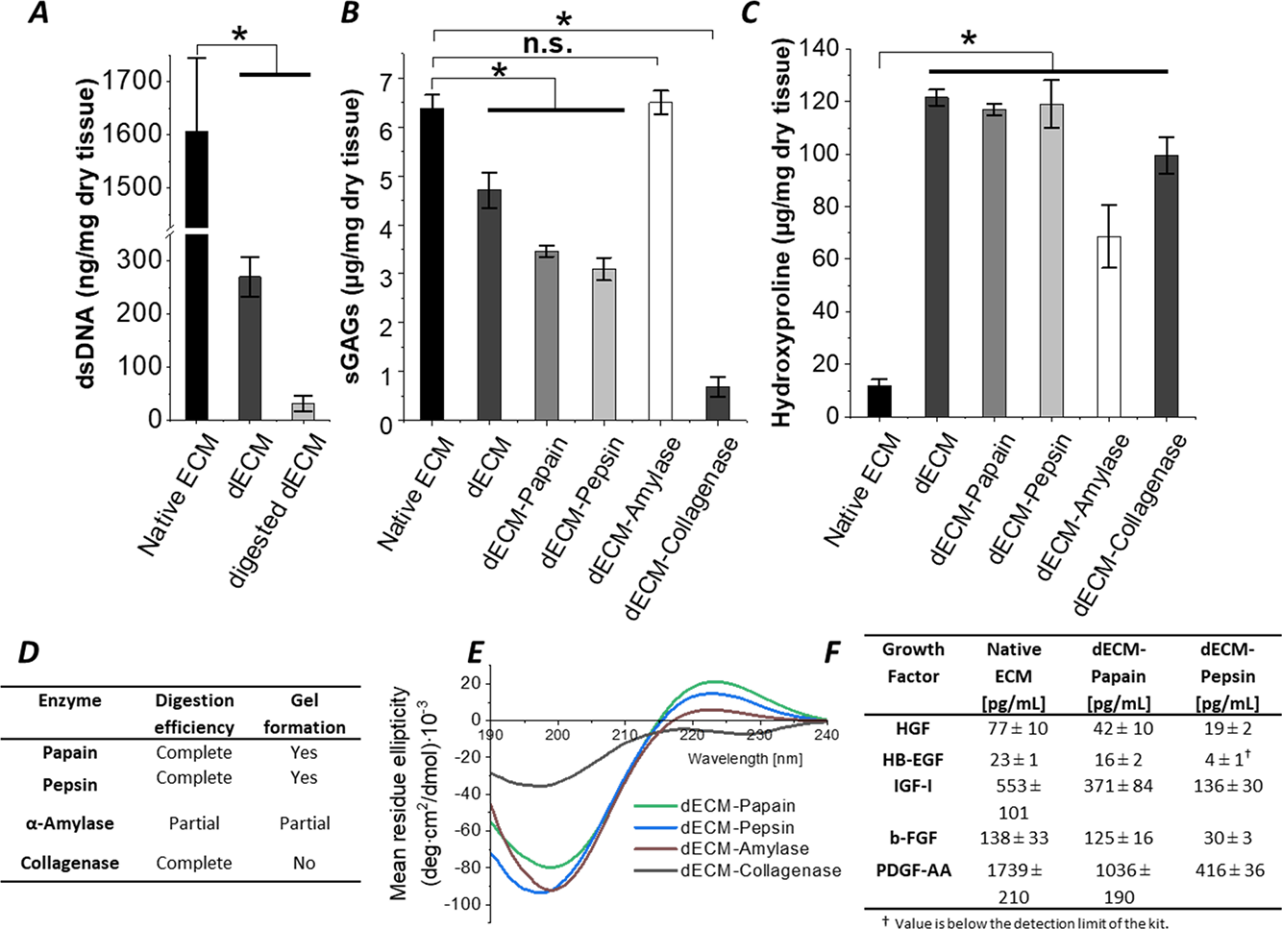
Biomolecular analysis of lyophilized native liver ECM, decellularized liver ECM, and its solubilized dECM digests obtained with different enzymes. (A) dsDNA content per mg dry weight (the dsDNA value for digested dECM is represented here by the value for dECM-Papain digests), (B) sGAG content, and (C) hydroxyproline content. (D) Qualitative digestion efficiency and gel formation capacity of resulting digests based on visual inspection and (E) CD-spectra of dECM digests at a concentration of 0.1 mg/mL in 7 mM phosphate buffer. (F) Growth factor and cytokine content of porcine native liver tissues compared to dECM samples digested with papain and pepsin assessed via a multiplex ELISA array. Data are presented as mean ± SD for *n* = 3, where * indicates statistical significance (*p* < 0.05) of all samples in comparison to native ECM according to a Kruskal–Wallis test followed by Dunn’s test.

The dsDNA content of the lyophilized undigested dECM was found to be 270 ± 37 ng/mg, while in all digested dECM, the dsDNA content was reduced below the accepted threshold limit of 50 ng/mg^1^, as representatively illustrated in Figure 2A for digests from papain. Colorimetric assays revealed the sGAG and hydroxyproline content of the solubilized dECM samples in comparison to decellularized and native ECM. The sGAG content decreased significantly in the tissues after cell removal and kept decreasing after digestion with different enzymes except for α-amylase (Figure 2B). In contrast, hydroxyproline, indicative of collagen content, was greatly enriched by tissue decellularization and maintained its level during digestion, except for a slight decrease in collagenase-solubilized samples and a notable reduction in α-amylase-treated dECM (Figure 2C).

The acidic pregel solutions were investigated for their gel-forming potential by visual inspection at a concentration of 10 mg/mL under physiological conditions (1x PBS (−/−), pH= 7.4 and 37 °C). Interestingly, only papain- and pepsin-digested dECM preparations formed stable gels after neutralization, while dECM-Collagenase digests did not form any gels at pH 7.4. In contrast, gel formation of α-amylase-digested dECM pregels was unreliable and resulted in unstable gels (Figures 2D and S2A).

To investigate the protein secondary structure of dECM digests, far-UV CD spectra of the buffered solutions were recorded. The diminishing positive band around 220 nm indicates that the collagen triple helical structure of dECM-Collagenase digests was destroyed, while the weak negative band around 200 nm suggests a partially random coil structure (Figure 2E). By contrast, the triple helix structure of collagen was strongly preserved for dECM-Pepsin and dECM-Papain digests and was only slightly present for dECM-Amylase, whereas fewer random coil structures were observed compared with dECM-Collagenase digests. Additional FTIR spectra showed similar and characteristic bands for collagen in both dry undigested and digested dECM, particularly for pepsin and papain digests.^31^ FTIR spectra and band assignments can be found in Figure S2B,C. As the collagenase and amylase-based dECM digests showed (partial) denaturation in the secondary structure of collagen (Figure 2E) and did not form stable hydrogels (Figure S1A), they were excluded from further in-depth comparative analysis.

Native ECM, as well as pepsin- and papain-digested dECM, was further evaluated for its growth factor and cytokine content via a multiplex ELISA array. In general, the dECM digests contained a wide variety of growth factors and cytokines, with some even in the ng/mL range, such as bone morphogenetic proteins 5 and -7 (BMP-5/-7), insulin-like growth factor-binding protein 3, -4, and -6 (IGFBP-3/-4/-6), insulin, platelet-derived growth factor AA (PDGF-AA), and transforming growth factor β1 and -β3 (TGF-β1/-β3) (Table S2), indicating potential bioactivity of the material. As a general trend, the growth factor levels of dECM decrease upon pepsin and papain digestion, while for most growth factors, a multiple of 2–3 is preserved for dECM-Papain compared to dECM-Pepsin digests. Importantly, growth factors such as hepatocyte growth factor (HGF), heparin-binding epidermal growth factor-like growth factor (HB-EGF), insulin-like growth factor 1 (IGF-1), basic fibroblast growth factor (b-FGF), and platelet-derived growth factor AA (PDGF-AA), which play a central role in liver regeneration, were also detected (Figure 2F, Table S3).^32^ The cytokine and growth factor levels in dECM-Amylase were also quantified, as shown in Table S2. Despite its low digestive efficiency, the growth factor and cytokine preservation were exceptionally high for α-amylase compared to papain and pepsin digests. Furthermore, endotoxin levels in the decellularized and digested ECM hydrogels were determined as described in the Supporting Information: 0.01 ± 0.004, 0.013 ± 0.006, and 0.011 ± 0.003 EU/mL for dECM, dECM-Papain, and dECM-Pepsin, respectively.

### 3.3 Molecular Mass Distribution of dECM and Proteomic Analysis

**Figure 3:**
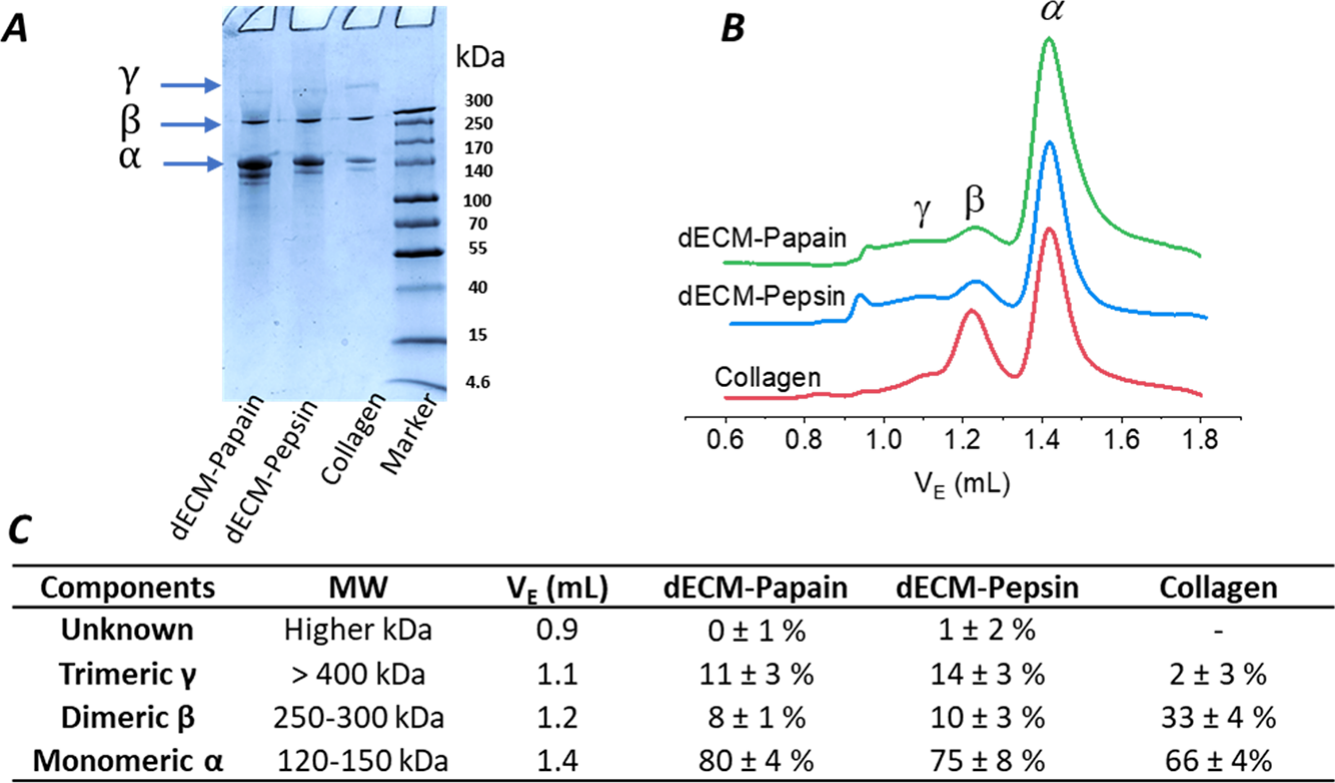
Molecular weight analysis of papain- and pepsin-digested dECM pregels compared to commercial collagen type I from rat tail. (A) Image of SDS-PAGE of dECM digests at 0.5 mg/mL and collagen at 0.1 mg/mL as a reference. (B) Semiquantitative SEC chromatograms of reduced and alkylated samples at 1 mg/mL concentration. (C) Table of relative peak areas of the α-, β-, and γ-chain fragments of collagen and higher molecular weight components in the SEC profiles of the samples. Data are presented as mean ± SD for *n* = 3.

The molecular mass distributions of dECM digests prepared using papain and pepsin were characterized with SDS-PAGE and SEC analysis. The stained SDS-PAGE image (Figure 3A) revealed that all collagen I-specific bands attributed to α-, β-, and γ-chains are present in both papain- and pepsin-digested dECM pregels. The monomeric α chains (1× α [I] and 2× α [II] chains) around 130 kDa appear most intense in the dECM-Papain preparation and expand to lower molecular ranges, indicating also smaller protein fragments compared with preparations of dECM-Pepsin. Fewer dimeric β-chains (250–300 kDa) and trimeric γ-chains (around 400 kDa) were detected in dECM-Papain compared to dECM-Pepsin pregels. The semiquantitative analysis of SEC traces (Figure 3B), which have been obtained after the reduction and alkylation of the samples, are summarized in Figure 3C, supporting the SDS-PAGE observations. In the chromatograms, the SEC profile of collagen type I is easily recognizable with the three main signals at elution volumes *V*_E_ of 1.4, 1.2, and 1.1 mL, corresponding to α-, β- and γ-chains, respectively.^33^ In general, the two dECM digests showed very similar profiles, with the collagen α-chain predominating over the β-chain signal, while a broad signal between 1 and 1.2 mL suggests the presence of γ-trimers along with higher mass proteins. To our knowledge, SEC analysis has never been reported for dECM digests, but only for ECM single components such as collagen and gelatin.^33^ Compared to collagen, the amount of dimeric β-chain fragments is reduced in both dECM digests, while an additional signal is observed at 0.9 mL, corresponding to even higher molecular weight components that could not be detected with SDS-PAGE. The relative abundance of each component was estimated from the deconvoluted SEC chromatograms and reported as the relative area (%) in Figure 3C.

**Figure 4:**
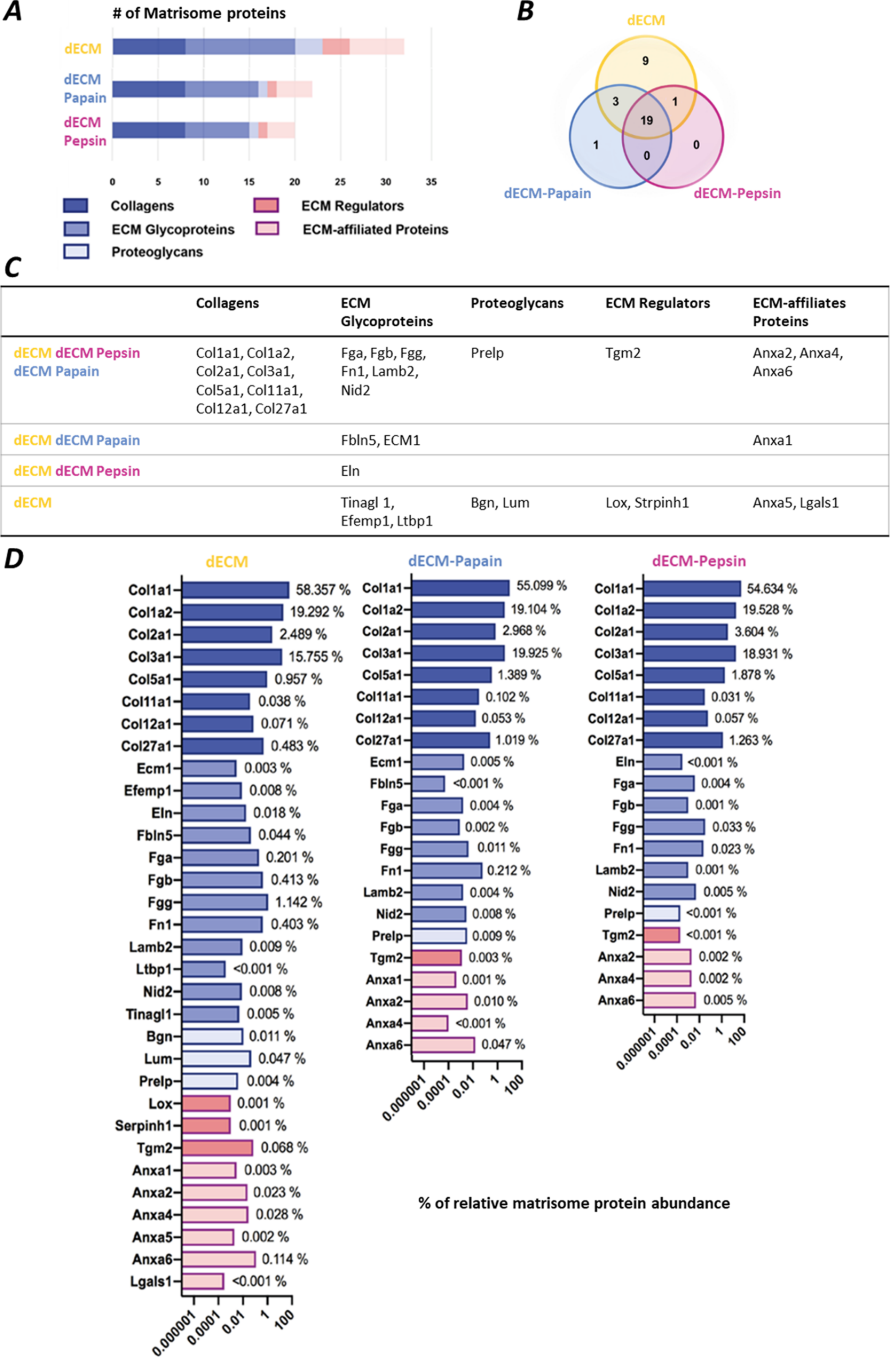
Principal component analysis of matrisome proteins present in dECM, dECM-Papain, and dECM-Pepsin. (A) Bar graph showing the number of matrisome proteins identified in different samples. (B) Venn diagram showing the proteomic composition overlaps among all groups. (C) Table showing the categorized groups according to Naba et al.^24,25^ (D) Bar graphs showing the percentage of relative matrisome protein abundances in different samples.

As expected, the concentrations of many other components in dECM pregels are too low to be detected via SEC. Therefore, the residual proteomic content after decellularization and digestion was characterized via mass spectrometry analysis. In the mass profiles (Figure 4A), 20 to 32 proteins were identified and categorized according to Naba et al.^24,25^ into matrisome protein subtypes, including collagens, ECM glycoproteins, proteoglycans, ECM regulators, and ECM-affiliated proteins. Overall, more core matrisome and matrisome-associated proteins were detected in dECM compared to digested dECM, and 19 matrisome proteins could be found in common among all groups (Figure 4B). The shared profiles of matrisome proteins between different groups are further sorted in a table and presented in Figure 4C. Even though undigested dECM showed a higher variety of proteins in all matrisome categories, collagens detected in the digested and undigested dECM samples were comparable in numbers and types, supporting the comparable digestive power of the used enzymes (Figure 4C,D).

In total, the collagen subtypes (COL1a1, COL1a2, COL2a1, COL3a1, COL5a1, COL11a1, COL12a1, and Col27a1) and ECM glycoproteins (Fga, Fgb, Fgg, Fn1, Lamb2, Nid2, Fbln5, Ecm1, Eln, Tinagl1, Efemp1, and Ltbp1) constituted about 97% of the total detected matrisome of all samples and thus represent the most abundant protein elements in the porcine liver extracellular matrix (Figure 4D). While collagens were similar in dECM-digests and undigested dECM, the numbers and abundance of ECM glycoproteins clearly varied and were reduced from undigested dECM over dECM-Papain to dECM-Pepsin digests. Both laminin (Lamb2) and fibronectin (Fn1) decreased after digestion by 50%- and >90% for both dECM-Papain and dECM-Pepsin, respectively. Furthermore, fibrinogen (Fga, Fgb, and Fgg) values similarly declined for both digests, while elastin (Eln) that existed in dECM was not found in dECM-Papain and was only present in dECM-Pepsin, although in low abundance. Finally, two core matrisome proteins (ecm1 and fibulin-5) and one matrisome-associated protein (annexin A1) were present in the undigested dECM and papain-digested samples but absent in the pepsin-digested sample (Figure 4D).

### 3.4 Gelation Kinetics and Rheological Characterization of Papain- and Pepsin-Digested dECM

**Figure 5:**
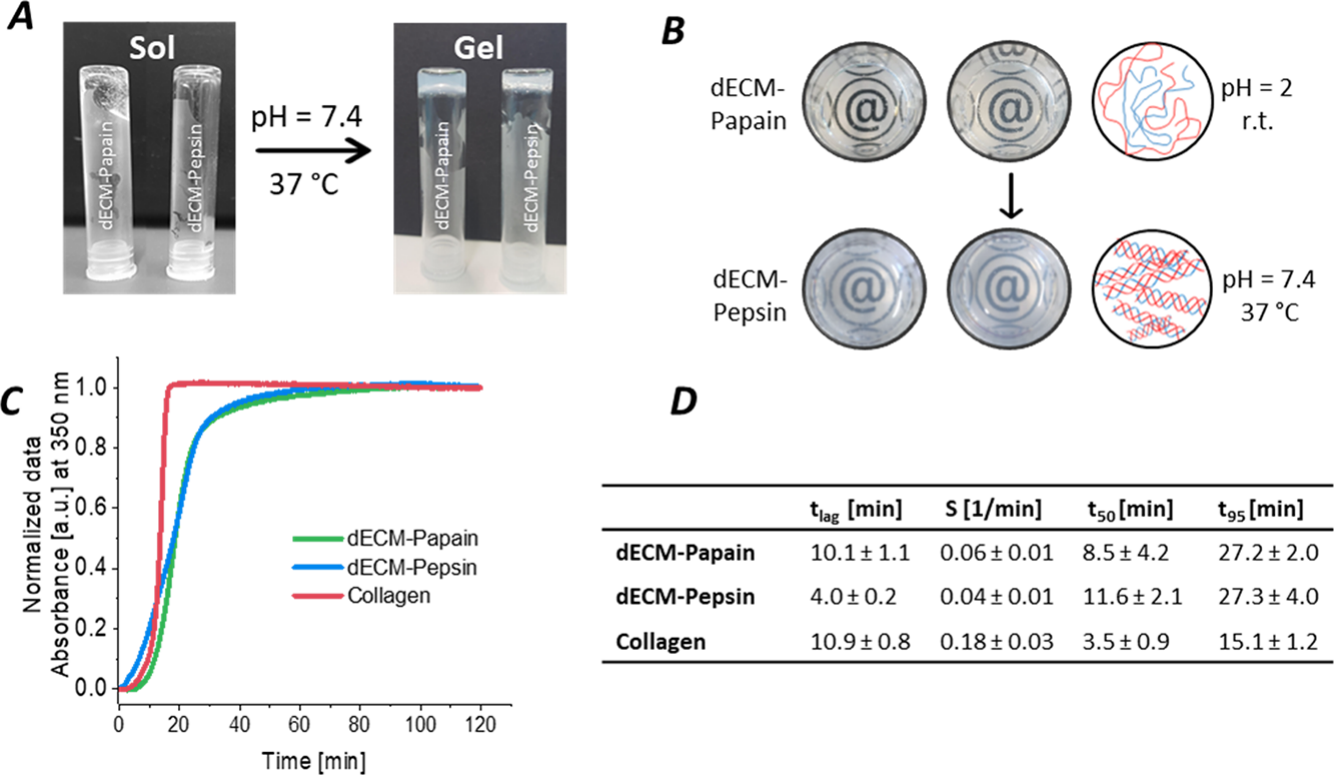
Gelation properties of dECM pregels (10 mg/mL) derived from pepsin and papain digestion compared to pure rat tail collagen (2.5 mg/mL). (A, B) Schematic illustration and photographs of the pH-induced sol–gel transition of dECM-Pepsin and dECM-Papain pregels via pH-induced self-assembly and cross-linking. (C) Normalized turbidity curves of pregels indicating the kinetics of fibrillogenesis induced by pH neutralization at 37 °C. (D) Characteristic fibrillogenesis parameters as mean ± SD for *n* = 3: Lag time *t*_lag_, slope S, time *t*_50_ and *t*_95_ to reach 50 and 95% turbidity, respectively.

In order to illustrate the sol–gel transition of the dECM hydrogels via self-assembly, photographs of the dECM-pregels at pH 2 at RT and neutralized gels at physiological conditions after 1 h of incubation at 37 °C were taken (Figures 5A,B and S2A). Both dECM-Papain and dECM-Pepsin pregels were transparent at acidic pH and exhibited a translucent appearance after gelling at 37 °C in neutral conditions, as indicated by the slightly reduced readability of the @-symbol. The turbidimetric gelation kinetics of dECM-Papain and dECM-Pepsin pregels were spectrophotometrically studied with stiffness-matched samples (see below) at a fixed wavelength of 350 nm at 10 mg/mL and compared to commercial rat-tail collagen type I at a concentration of 2.5 mg/mL, which has been reported to be an optimal collagen concentration for cell-laden hydrogels elsewhere.^34,35^ The detection principle is based on the increase in turbidity resulting from collagen self-assembly. The normalized, time-dependent absorbance curves for dECM-Papain, dECM-Pepsin, and collagen are shown in Figure 5C, which exhibit a sigmoidal profile, with the dECM samples gelling after a longer lag phase than pure rat tail collagen. The related gelation parameters lag time *t*_lag_, slope S, time to 50% gelation *t*_50,_ and time to reach 95% maximum turbidity *t*_95_ were calculated as presented in Figure 5C. dECM-Pepsin pregels showed the fastest initiation of gelation (*t*_lag_ = 4 ± 0.2 min), while dECM-Papain pregels had a longer lag time (*t*_lag_ = 10.9 ± 0.8 min) comparable to pure collagen samples (*t*_lag_ = 10.1 ± 1.1 min). From analyzing the slope values, which indicate the gelation speed, pure rat tail collagen samples surpassed the digested samples by a factor of 2 to 2.5. The time to reach 50% gelation located at *t*_50_ = 3.5 ± 0.9 min for pure collagen and *t*_50_ = 8.5 ± 4.2 and 11.6 ± 2.1 min for dECM-Papain and dECM-Pepsin, respectively. The digested dECM samples reached 95% gelation similarly in *t*_95_ ∼ 27 min, whereas pure rat tail collagen samples required only *t*_95_ ∼ 15 min.

**Figure 6:**
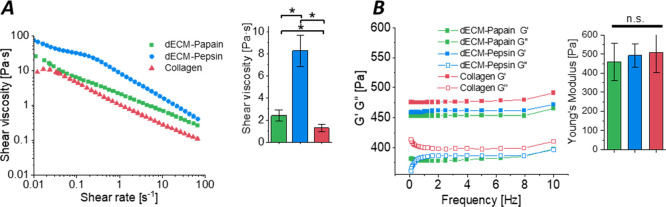
Rheological properties of dECM hydrogels (10 mg/mL) derived after papain and pepsin digestion compared to those of pure rat tail collagen (2.5 mg/mL). (A) Representative complex viscosity curves accessed with a rotational rheometer and their respective plotted values (mean ± SD, *n* = 3) at a shear rate of 1 s^–1^. (B) Representative storage (*G*′) and loss modulus (*G*″) curves of the gels over a shear range of 0–10 Hz and the resulting values at a frequency of 5 Hz represented as a bar graph (mean ± SD, *n* = 3). *Indicates statistical significance (*p* < 0.05) as evaluated by a Kruskal–Wallis followed by Dunn’s test.

The shear flow viscosity of neutralized pregels was measured before gelation at 15 °C under a shear rate ranging from 0.01 to 100 s^–1^. All samples showed shear-thinning behavior and the values were approximately 1, 2, and 8 Pa·s at a shear rate of 1 s^–1^ for rat tail collagen, dECM-Papain, and dECM-Pepsin pregels, respectively (Figure 6A). The dynamic storage modulus *G*′ and loss modulus *G*″ of all gelled samples were investigated at physiologically relevant temperatures to ensure their stability after the 2-hour gelation process. The storage modulus remained stable under the frequency sweep, and the gels revealed no significant difference in their stiffness at 5 Hz (Figure 6B).

### 3.5 Ultrastructural Characterization, Surface Morphology, and Surface Micromechanical Properties

**Figure 7:**
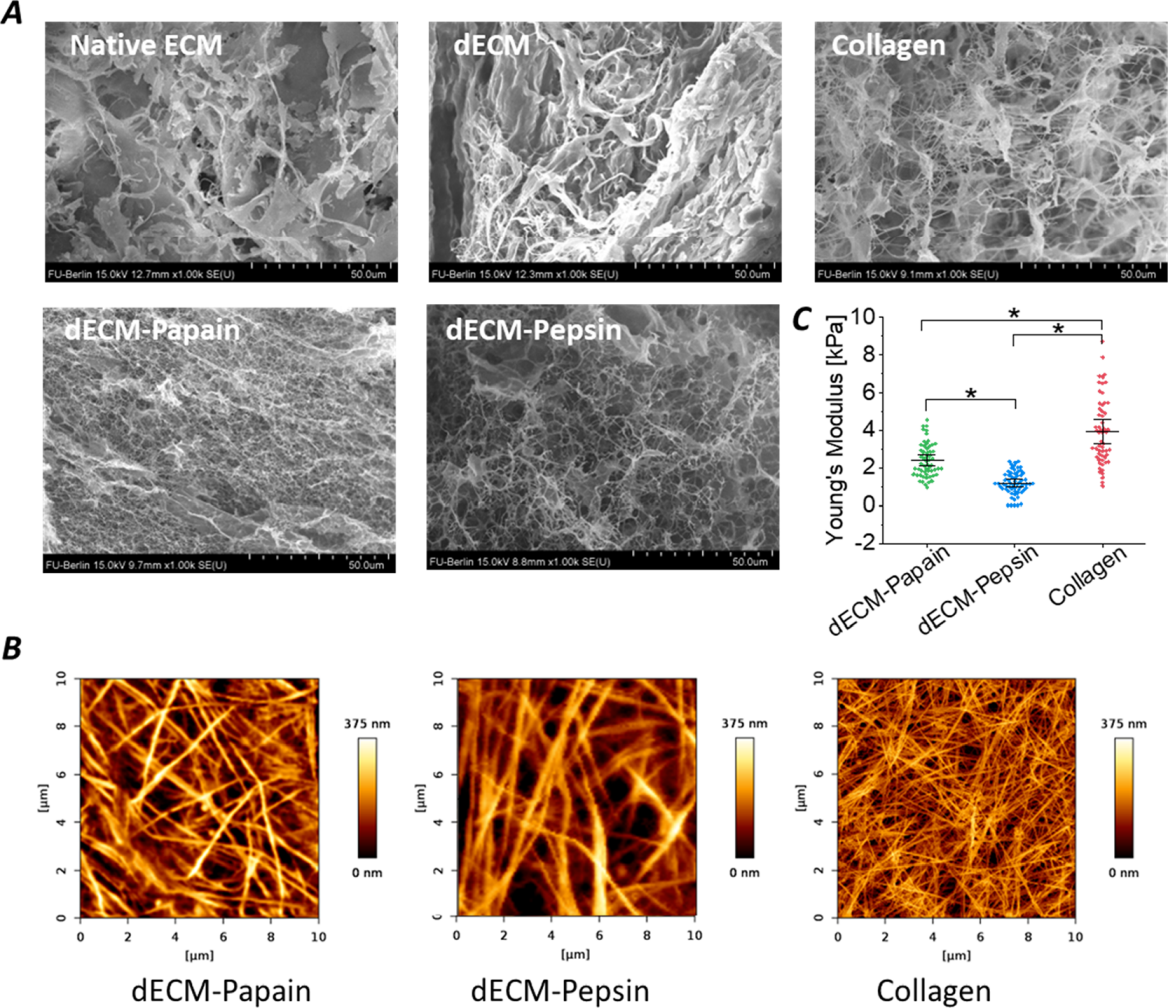
Characterization of the matrix structure and local stiffness of digested dECM (10 mg/mL in 1× PBS (−/−)) and collagen (2.5 mg/mL in 1× PBS (−/−)) gels. (A) Representative SEM images of the inner gel structure of lyophilized hydrogels and native porcine liver ECM and dECM as controls (scale bar = 50 μm). (B) Representative surface morphology of the wet hydrogels accessed via AFM at 37 °C in 1x PBS (−/−). (C) Local Young’s modulus of the wet hydrogel surfaces accessed via AFM nanoindentation at 37 °C. Data are presented as mean and whiskers with a 95% confidence interval (*n* = 64–100 curves). * Indicates statistical significance (*p* < 0.05) confirmed by one-way ANOVA followed by Tukey’s test.

The internal ultrastructures of lyophilized samples derived from digested dECM or collagen hydrogels as well as native ECM and dECM were visualized by SEM after sputtering to infer their network structure. In Figure 7A, the sheet-like and thick bundle shape is present in both lyophilized porcine liver ECM and dECM tissues. Interestingly, after digestion with either papain or pepsin, the hydrogels appeared as a highly branched and woven mesh. Visual hydrogel interconnectivity was highest for the dECM-Papain hydrogels and slightly decreased in the dECM-Pepsin hydrogels, while it clearly differed for the pure collagen type I hydrogels for which both fibrous and sheet-like structures were observed.

The morphology of the hydrated gels was studied on the nano- and microscale by scanning AFM, which enabled a better evaluation of their fibrous network. The morphological images of papain and pepsin-digested dECM hydrogels at a concentration of 10 mg/mL, as well as images of collagen controls at 2.5 mg/mL in PBS (−/−) buffer, are presented in Figure 7B. While the pure rat tail collagen showed a very dense fibrillar structure with very thin and short fibers, the length and thickness of fibers increased for dECM-Papain samples with a density similar to collagen. The dECM-Pepsin samples exhibited even longer and thicker fibers but with a lower network density than the other two samples. To our knowledge, there are no previous reports on the surface morphology of widely used dECM-Pepsin hydrogels. Further morphological images can be found in Figure S2.

The respective nanoscale mechanical properties of the gel surfaces were further evaluated by AFM nanoindentation measurements. Several force–distance curves were collected from each surface on 64 different nano sites. The AFM analysis of the local stiffness on the wet hydrogel surfaces revealed Young’s moduli in the low kPa ranges of 1.2 ± 0.6 (dECM-Pepsin, 10 mg/mL), 2.5 ± 0.89 (dECM-Papain, 10 mg/mL), and 4.0 ± 1.7 kPa (collagen, 2.5 mg/mL) (Figure 7C). Furthermore, we quantified the nanoscale roughness by the root-mean-square (RMS) surface roughness of the hydrogels from AFM images taken with a sharp tip (radius 2 nm), showing that pure commercial collagen had the lowest surface roughness (50 ± 7 nm), which was slightly increased for both dECM-Papain and dECM-Pepsin with 63 ± 5 nm and 71 ± 8 nm, respectively. Therefore, the surface roughness increased with the decreasing nanoscale surface elastic modulus.

### 3.6 Sterilization and Cytocompatibility of dECM-Derived Hydrogels

**Figure 8:**
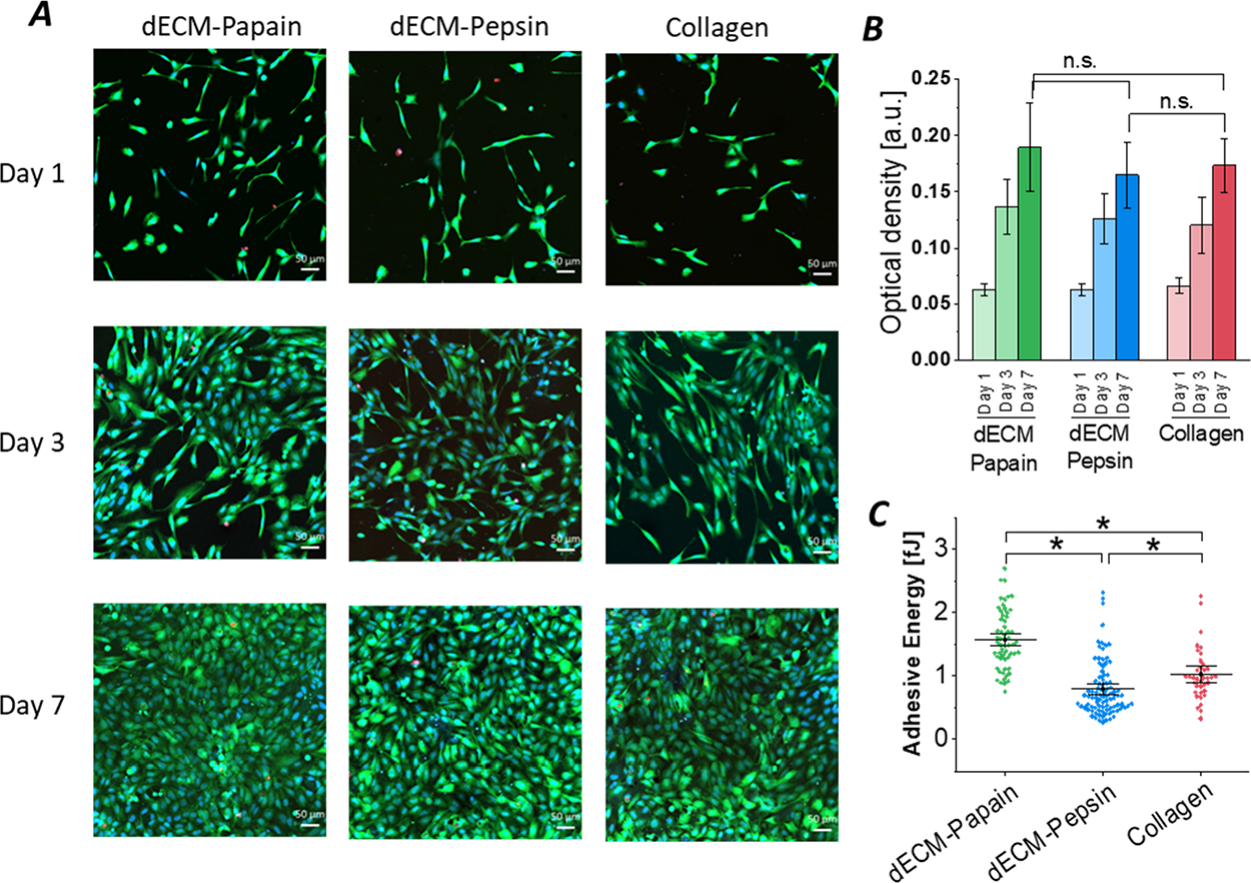
Cytocompatibility assessment of pepsin and papain digested liver dECM hydrogels (10 mg/mL) in comparison to rat collagen type I (2.5 mg/mL) as a control. (A) Representative confocal fluorescent images of human HepaRG cells seeded on the gel surface after fluorescent staining with Hoechst (blue, nuclei) with FDA (green, live cells) and PI (red, dead cells) on days 1, 3, and 7 in culture (scale bar = 50 μm). (B) Time-dependent metabolic activity of human HepaRG cells accessed via a Presto Blue assay (mean ± SD, *n* = 3). Statistical analysis was performed by the Kruskal–Wallis test followed by Dunn’s test. (C) Single-cell adhesion forces between human fibroblasts and hydrogel surfaces measured by AFM force spectroscopy are shown as mean and whiskers with 95% confidence interval (*n* = 60–100 curves). Statistical analysis was performed by one-way ANOVA followed by Tukey’s test. * Indicates statistical significance (*p* < 0.05).

To prepare the hydrogels for cell culture, the dECM pregels were successfully sterilized via dialysis in either 0.1% of peracetic acid or 1% chloroform for 1 hour, followed by dialysis in sterile diluted acid.^36^ The cell compatibility of the gels was tested by seeding human HepaRG cells on the surface of dECM hydrogels and collagen controls at a seeding density of 27 × 10^3^ cells/cm^2^ and continuing the culture for 7 days. Fluorescent live/dead staining on days 1, 3, and 7 of the culture (Figure 8A) revealed similar adhesion, proliferation, and viability of the human liver cells on the dECM hydrogels compared with cultures on the pure rat tail collagen gels. The metabolic activity of HepaRG cells cultured on the hydrogels was followed by a colorimetric PrestoBlue assay (Figure 8B). A continuous and comparable increase in the mitochondrial cell metabolism over the time course of 7 days was detected on all hydrogels, being the highest on papain-dECM hydrogels on days 3 and 7. Additional single-cell force spectroscopy measurements with human fibroblasts at 37 °C in PBS (+/+) enabled a closer look at the initial contact and adhesion forces between a single cell and the hydrogel substrates (Figure 8C). The measurements indicated significantly higher initial fibroblast adhesion to dECM-Papain gels than dECM-Pepsin and collagen gels.

## 4 Discussion

For large-scale production of dECM hydrogels, the cost, stability, and efficiency of enzymes are the main factors determining the economics of the solubilization process. Therefore, various enzymes of different origins need to be evaluated for their solubilization efficiency and the biophysical and biochemical properties of the resulting materials. In our current work, we decellularized porcine liver tissues via a chemical treatment involving anionic SDS and nonionic Triton X-100 surfactants (Figure 1B). This treatment removed dsDNA significantly (∼83%), although the amount was still above the generally accepted threshold limit (<50 ng/mg).^1^ Subsequent digestion and purification further reduced the dSDNA content to an acceptable value of 32 ± 14 ng/mg (Figure 2A). The enzymatic cleavage in the dECM during digestion facilitated the release of the residual digested DNA, which could thus be removed via dialysis (in 0.01 M HCl), thereby highlighting the importance of the final purification step. Furthermore, endotoxin levels were assessed, revealing very low concentrations (around 0.01 EU/ml) in all samples. This was well below the FDA standards (0.5 EU/mL for biologic scaffold eluates),^37^ highlighting their general suitability for use in tissue engineering and regenerative medicine. However, their specific safe use in terms of a potential adverse immune response in vivo particularly in the long term during and after cellular matrix remodeling needs to be assessed. There is currently no specific guidance from the FDA regarding remnant cellular content in commercially available decellularized tissues, while a number of yet inconclusive data has been reported on the immune (in)tolerance of dECM-based materials, largely varying with the tissue type, source, and processing.^38^

Although α-amylase does not fall under the category of classical peptide proteases, it was included in this study based on recent research demonstrating its efficacy as a digestive enzyme for successfully generating dECM hydrogels.^12,13,15^ Based on the enzyme manufacturers’ recommendations for buffers, pH, and temperature, the digestion time, steps, and enzyme concentration were optimized for all tested enzymes via visual inspection of the digestion progress (Figure S1). Similar to animal-derived pepsin, plant-derived papain yielded fully solubilized dECM within 48 h of digestion. This time was further set as a benchmark in the evaluation of other enzymes since shorter digestion times with pepsin can result in inhomogeneous mixtures, whereas longer times (>72 h) have shown a decrease in the viscosity and stiffness of the final hydrogels with impaired cell compatibility.^11^ While collagenase digested the dECM completely by a continuous cleavage of the protein bonds and ended up with low viscous solutions with a huge reduction in yields down to ∼7% after purification, α-amylase required an additional second step of acidic extraction for dECM solubilization. Shorter digestion times with collagenase did not change the viscosity of the resulting protein fragments and resulted in only more undigested dECM chunks, while longer enzyme treatment times with α-amylase (or a higher enzyme concentration) showed no improvement in the efficiency even after 7 days of digestion, indicating that the α-1,4 glycosidic bonds have been cleaved already, leaving behind mainly undigested collagen (Figure S1). However, collagen solubility in slightly acidic conditions is enhanced after α-amylase treatment due to destabilized collagen-glycoprotein complexes and cleaved carbohydrate groups in the telopeptide regions of collagen.^39,40^ Unlike other studies where bacterial-derived α-amylase (activity: 30 U/mg and 0.3% w/w of dry dECM)^41^ was successfully used to digest decellularized adipose tissue^15^ and decellularized left ventricle,^42^ digestion of porcine liver tissues with α-amylase yielded in our study only low amounts of soluble proteins (10.1 ± 2.7% yield) with impaired ability to form a hydrogel at neutral pH (Table 2 and Figure S2A). This indicates the importance of the tissue source for efficient digestion with a distinct enzyme.

In contrast to α-amylase, proteolytic papain and pepsin proved highly efficient in digesting liver-derived dECM with isolated yields of 79 ± 5 and 86 ± 6% after purification, respectively (Table 2). Papain cleaves collagen’s terminal nonhelical telopeptide regions from the two α1 chains at the same site and the α2 chain at different sites compared to pepsin.^43^ Therefore, it can facilitate the lift of the intact soluble helical region without affecting its bioactive properties, as demonstrated with enzymatic collagen extracts from several animals’ skin and feet.^14,44−46^ Advantageous characteristics over acid-extracted collagen, such as high protein solubility and higher yields, have been observed, while the thermal stability of the resulting collagen was not affected.^43^ Despite this, using papain in dECM digestion for biomedical applications is rare, except for preparing dECM hydrolysates under harsh conditions, allowing the quantitative detection of DNA, sGAG, and hydroxyproline content.^47,48^ While the pH-induced gelling potential of papain digests has not been previously shown, some bioactive properties of papain-digested, short-chained dECM fragments have been reported, such as chemotactic and mitogenic effects on endothelial progenitor cells.^49,50^

The performed biochemical characterization of all ECM-digests showed a reduction in its sGAG content, except for dECM-Amylase, and enrichment in its hydroxyproline content compared to native ECM, as reported elsewhere (Figure 2B,C).^51^ Interestingly, dECM-Amylase revealed a preserved level of sGAG compared to the native-ECM and a lower hydroxyproline content compared to those of other enzyme digests. This can be attributed to the nonproteolytic nature of α-amylase, hinting toward a different compositional profile of dECM-Amylase compared to other proteolytic digests. Only dECM-Papain and dECM-Pepsin pregels were able to form hydrogels upon incubation at physiological conditions (Figure 5A,B), while dECM-Amylase digests formed unstable and inhomogeneous gels and dECM-Collagenase did not self-assemble into any gel structures (Figure S2A). This visual observation was further supported by the CD analysis (Figure 2E) of the protein secondary structure within the dECM pregels, mainly composed of collagen. Among all enzymes, collagenase had the most detrimental effect on the proteins’ structure, destroying completely the α-helices.^52^ While the other proteolytic enzymes digest collagen on either its terminal telopeptides’ side chains or the glycosidic bonds, collagenase splits the α chains of the triple helix directly into fragments.^53^ In contrast, dECM-Amylase pregels indicated partial denaturation, while dECM prepared by papain and pepsin digestion were very similar in their undenatured profiles.

Interestingly, all dECM-digests that formed stable hydrogels could also preserve various growth factors and cytokines (Figure 2E and Table S2). Of particular interest was the presence of HGF, EGFR and HB-EGF, TGF-α and -β, and bFGF and FGF, members of the vascular endothelial growth factor (VEGF)- and IGF-family as well as insulin in the dECM digests.^32,54^ All these growth factors are important for stimulating cell growth, proliferation, and differentiation in the liver, particularly HGF. In a recent report by Ijima et al., the concentration of HGF was enhanced beyond the inherent HGF levels already present in the dECM by supplementing both dECM and collagen control with an additional 10–100 ng/mL of HGF.^55^ When a liver dECM hydrogel was implanted into a partial hepatectomy rat model with encapsulated hepatocytes, the number of viable hepatocyte clusters was significantly higher for dECM versus collagen after 1 week. Their efficiencies of liver-specific ethoxyresorufin-O-deethylase (EROD) activity and large liver-tissue-like structure formation were about twice those of collagen gel-embedded hepatocytes. The preservation of dECM-related growth factors and cytokines after pepsin digestion and chemical modifications has been reported for many tissue types.^56−60^ In this study, most growth factors are preserved in higher quantities for papain than pepsin digests and located in the same order of magnitude as detected in the originating native ECM. Notably for α-amylase digests, a clear increase in growth factor preservation compared to papain and pepsin digests and occasionally even an enrichment compared to native ECM was observed (Table S2). The latter might be an effect of the nonproteolytic, glycosidic activity of α-amylase retaining growth factors intact and releasing them from GAGs. The ability of the liver dECM to immobilize growth factors due to the presence of GAGs compared to acid-extracted collagen has been reported.^61^ In summary, despite the compelling indications of the existence of growth factors within the dECM-Papain and dECM-Pepsin digests, additional experimentation and validation are needed to determine the potential benefits of their biological activity on cellular function and response.

Looking closer at the protein composition, SDS-PAGE and SEC analysis revealed a similar pattern of the molecular mass distribution of dECM-Papain and dECM-Pepsin compared to that of rat tail collagen type I, including all characteristic collagen α, β, and γ bands (Figure 3A,B). Despite this, the ratio of monomeric α to the sum of dimeric β and trimeric γ units was 4.2 for papain and 3 for pepsin, while collagen exhibited a ratio of 1.8. In addition, the SEC signal of the unknown higher molecular weight species was the largest in the pepsin digest profile (Figure 3C). Overall, these results suggested that papain led to approximately 80% monomeric α units, while pepsin digestion resulted only in ∼75% α units on average. As the ratio of high and low molecular weight species in the dECM digest can have a direct impact on the collagen self-assembly similar to the presence of GAGs and other (glyco)proteins, we tested their fibrillogenesis kinetics that revealed a sigmoidal profile for both dECM-digests. This was in good agreement with previous studies on dECM-Pepsin.^62,63^ While the initiation of gel formation was fastest with dECM-Pepsin (10 mg/mL) after neutralization, it was slightly slower for dECM-Papain (10 mg/mL) but still comparable to that of pure collagen (Figure 5A–C). This might be explained by the presence of 1 ± 2% higher molecular weight molecules (>500 kDa) in the dECM-Pepsin in comparison to only 0 ± 1% in the dECM-Papain and none in pure collagen, as detected in the SEC profiles (Figure 3B,C). Even though the dECM-Pepsin digest was the first to initiate the self-assembly process, the time needed to reach 95% gelation was similar for the dECM-Papain pregel (*t*_95_ ∼ 27 min), with both of them being slower than the pure collagen pregel (*t*_95_ ∼ 15 min). This is in general agreement to literature, where a slower gelation growth phase has been reported for pepsin-digested cornea dECM (16 mg/mL) and urinary bladder dECM (6 mg/mL) compared to rat tail collagen (3.0–3.5 mg/mL).^63,64^ For most applications, the gelation kinetics of the dECM pregels are still within a well-practicable range and might even be advantageous compared to fast-gelling collagen, allowing more time for handling.

Even though collagen is the major constituent of ECM and hence also solubilized dECM, the decellularization and solubilization method, for example, acid- vs enzyme-extracted collagen, can greatly impact the gelation properties. This can be explained by the presence of telopeptides in the acid-extracted collagen that promotes the self-assembly of collagen molecules, resulting in a higher fibrillogenesis rate, unlike the pepsin-extracted collagen where the telopeptides region has been removed during the digestion process.^65^ Since the mass distribution analysis (SEC profiles) revealed different profiles for the dECM digests compared to the pure rat tail collagen, especially in the very high and very low molecular weight regions, the flow behavior was studied by rheology. All pregels showed a shear thinning behavior, and agreeing with its largest high molecular weight fraction, the dECM-Pepsin digest (10 mg/mL) exhibited the highest viscosity compared to the dECM-Papain (10 mg/mL) digest and pure rat-tail collagen (2.5 mg/mL) (Figure 6A). This is particularly relevant for 3D bioprinting applications of the dECM gels, where the rheological profile of the bioinks is essential.^51^ The macro mechanical analysis with the oscillating rheometer showed similar stiffness (∼500 Pa) for both dECM hydrogels (10 mg/mL) and collagen (2.5 mg/mL) (Figure 6B), which is in good agreement with a previous report showing equivalent stiffness for a porcine liver-derived dECM-Pepsin gel (10 mg/mL) and a pure collagen gel (3 mg/mL).^66^ SEM images of the established lyophilized dECM hydrogels revealed an inner porosity that resembled a highly branched and woven mesh of fibers in contrast to the aggregated and bundle-shaped native ECM or undigested dECM (Figure 7A). Additional AFM studies of the surface morphology in the hydrated state (Figure 7B) displayed a dense fibrillar structure for all hydrogels. In combination with the SEC profiles, the images suggest that the hydrogels with the highest molecular weight proportion (dECM-Pepsin) showed the longest and thickest fibrils, while the hydrogels with no high molecular weight content (collagen) exhibited the shortest and thinnest fibrils. The results obtained from both AFM and SEC are consistent with the shear viscosity of the pregels (Figure 6A). The shear viscosity is directly influenced by the molecular weight and the concentration, or more fundamentally, by the extent of fiber chain entanglements.^67,68^ Notably, the pregels composed of dECM-Pepsin exhibited substantially higher shear viscosity due to the larger molecular weight fragments and longer fiber lengths than the pregels comprising dECM-Papain and collagen. The local stiffness of the hydrogel surfaces at the nanoscale increased with fibril density, resulting in the dECM-Papain gel (∼2.4 kPa) being significantly stiffer than the dECM-Pepsin gel (∼1.2 kPa) (Figure 7C). While this result deviates from a previous report where different subareas of the cell-populated articular cartilage tissue samples with thicker microfibrils and, therefore, lower density had a higher elastic modulus, the fibril density played a major role in tuning the nanomechanical behavior.^69^ The detected difference in the nanoscale stiffness of the dECM hydrogels did not influence the adhesion and growth of human HepaRG cells on the gel surface, as the cells showed similar surface coverage, viability, and indifferent metabolic activity compared to collagen gels on day 7 of culture (Figure 8A,B).

Our extensive proteomic analysis (Figure 4) of the dECM-Papain digest in comparison to pepsin-digested dECM and undigested ECM confirmed that the major ECM components were preserved with great overlap between the three samples. The main collagen isoforms, but also proteoglycans and ECM regulators, were preserved, which may play a role in the signal transduction of the cells cultured on and in the gel.^23^ Many exosomal proteins were also maintained within the decellularized matrix after digestion, including proteins related to cell adhesion (such as fibronectin Fn1), which mediate cell-matrix interactions at the cellular level.^70^ The larger amounts of fibronectin left in dECM-Papain (10× more compared with dECM-Pepsin) after digestion can provide more adhesion sites for cell interaction. This result correlates well with our single-cell adhesion studies on hydrogels, in which dECM-bound fibronectin also enhanced integrin-mediated adhesion energy to fibroblasts by ∼ 1-fold for dECM-Papain gels (1.6 fJ) compared to dECM-Pepsin (0.8 fJ) and collagen gels (1.0 fJ) after only 60 s of cell-matrix contact time (Figure 8C). The measured single-cell forces are well within the range of the recently reported initial-phase adhesion forces of single L929 fibroblasts to fibronectin-coated surfaces. This study also demonstrated higher adhesion energies with increasing contact times of 1, 5, and 30 s that ranged between 0.5 and 3 fJ.^71^ While contact times <60 s might seem too short to study such interactions, Strohmeyer et al. impressively showed that fibronectin-bound integrins can sense the mechanical load and signal to reinforce adhesion to fibroblast cells in less than a second.^72^ Overall, strong hints for enhanced bioactivity of dECM-Papain compared to dECM-Pepsin digests have been obtained, which require further validation.

## 5 Conclusions

In this work, we demonstrated that plant-derived papain is a highly attractive alternative to animal-derived pepsin for the straightforward, reproducible, and cost-effective production of liver-derived dECM hydrogels for tissue engineering and regenerative medicine. The simplicity of the dECM digestion and the low cost of the enzyme enabled the large-scale production of soluble dECM with comparable and, in terms of preserved potential bioactive molecules, even superior properties to pepsin digests for hydrogel preparations. As α-amylase-digested dECM showed the highest growth factor content, blending it with papain-digested dECM might offer a powerful strategy for preparing reproducible dECM hydrogels with enhanced bioactivity. However, it should be remembered that the dECM properties reported in this study are valid only within the limits of the dECM tissue type and processing parameters chosen here as they can affect dECM-based material properties significantly. Besides their economic attractiveness, plant-based enzymes can safely be applied to digest human extracellular matrices without the risk of zoonotic disease transmission. In our ongoing work, we successfully applied the new papain digestion protocol to porcine intestine, porcine lung, and human lung tissue with slight adaptations to accommodate the specific material properties (unpublished data), highlighting its great versatility across different tissue types. In the future, we will further elucidate the indicated bioactivity of these materials in more complex in vitro tissue models.

## Supporting Information Available

Additional experimental details, methods, and photographs of initial enzyme concentration screening for solubilization of porcine liver dECM, compilation of commercial enzyme costs as accessed and evaluated on 12/2022, FTIR spectra, growth factor content, and morphological AFM images of digested dECM (PDF)
