# Supplementary material for: Papain-Based Solubilization of Decellularized Extracellular Matrix for the Preparation of Bioactive, Thermosensitive Pregels
Source: Biomacromolecules. 2023 Nov 27;24(12):5620–37. doi: 10.1021/acs.biomac.3c00602 (PMC10716854; doi:10.1021/acs.biomac.3c00602)
Supplement: Supplementary file 1 — bm3c00602_si_001.pdf [file bm3c00602_si_001.pdf]

Papain-Based Solubilization of Decellularized Extracellular Matrix for the Preparation of Bioactive, Thermosensitive Pregels

*Ahed Almalla,[1] Laura Elomaa,[1] Leïla Bechtella,[1] Assal Daneshgar,[2] Prabhu Yavvari,[1] Zeinab Mahfouz,[1] Peter Tang,[2] Beate Koksch,[1] Igor Sauer,[2,3] Kevin Pagel,[1,4] Karl Herbert Hillebrandt,[2,5] Marie Weinhart [1,3,6,]**

[1]Institute of Chemistry and Biochemistry, Freie Universität Berlin, 14195 Berlin, Germany

[2]Experimental Surgery, Department of Surgery, CCM|CVK, Charité – Universitätsmedizin Berlin, Augustenburger Platz 1, 13353 Berlin, Germany

[3]Cluster of Excellence Matters of Activity. Image Space Material funded by the Deutsche Forschungsgemeinschaft (DFG, German Research Foundation) under Germany´s Excellence Strategy – EXC 2025

[4] Fritz Haber Institute of the Max Planck Society, Faradayweg 4-6, 14195 Berlin, Germany

[5]Berlin Institute of Health at Charité – Universitätsmedizin Berlin, BIH Biomedical Innovation Academy, BIH Charité, Clinician Scientist Program, Charitéplatz 1, 10117 Berlin, Germany

[6]Institute of Physical Chemistry and Electrochemistry, Leibniz Universität Hannover, 30167 Hannover, Germany

* Corresponding author: marie.weinhart@fu-berlin.de and marie.weinhart@pci.uni-hannover.de

Supporting Information

1.1. Experimental Section

Chromogenic Endotoxin Quantification

To test for the potential presence of endotoxins in dECM and dECM-digested hydrogels, lyophilized, foam-like samples (10 mg) were placed in cold PBS (−/−) at 4 °C for 24 h until they were soaked completely and a visual hydrogel was formed. Samples were disinfected by a 30 min treatment with 70% ethanol before washing them with sterile PBS (3 x 10 min) and equilibrating them overnight in sterile PBS (200 µL). The endotoxin assay was performed using a Pierce™ LAL chromogenic endotoxin quantitation kit according to the manufacturer's instructions.

1.2. Additional Figures

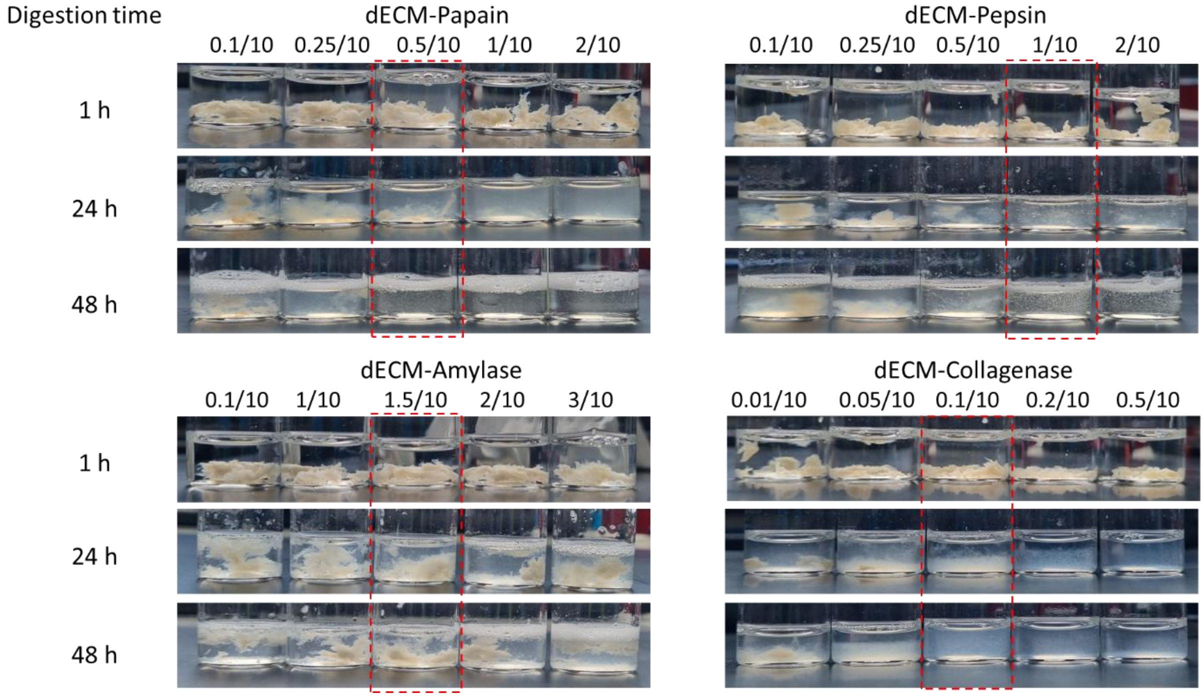

Figure S1. Representative photographs of the initial screening and preparation of porcine liver dECM-based digest after 1, 24, and 48 hours of digestion. The enzyme solutions were prepared according to Table 1 with the lyophilized dECM at a fixed 10 mg/mL dECM concentration and different enzyme-to-dECM ratios as stated on top of the picture columns and agitated constantly for 24 h at either 25 or 37 °C as specified in Table 1. After 24 h of digestion with α-amylase, the enzyme was inactivated by shifting the pH to acidic conditions (pH =2) on ice for 1 hour. Subsequently, the dECM-Amylase digest was allowed to stir for another 24 h at pH=2 as a second non-enzymatic step of protein solubilization. Papain, pepsin, and collagenase one-step digests were inactivated on ice after 48 h reaction time by changing the pH for 1 h to 9.5-10 for papain, pH 9

for pepsin, and pH 2 for collagenase. All samples were periodically homogenized using an IKA Ultra-Turrax® mixer during the digestion. Final images at 48 hours are obtained after enzyme deactivation. Red frames indicate the optimized enzyme-to-dECM ratio used throughout the manuscript.

Table S1. Description of enzymes price range from different suppliers and the costs of the exact enzymes used in this study.

Enzyme	Prices according to the CAS DataBase [a]	Price per 10 g of the exact enzyme used in this study [b]	Cost of an enzyme to digest 1 g liver dECM [c]
Papain	48 – 2970 $	16.9 € Sigma Aldrich/Merck (#1.07144)	0.08 €
Pepsin	23 – 9080 $	1280 € Sigma Aldrich (#P7012)	12.8 €
□-Amylase	14 – 2260 $	21.1 € MB biomaterials (#9000-90-2)	0.30 €
Collagenase	53 – 5570 $	573 € Sigma Aldrich (#C2-22-BIOC)	0.57 €

a. Cost range includes at least three different suppliers neglecting purity and activity as provided by *www.chemicalbook.com* and accessed on November 16, 2022; **b.** Prices as accessed online from the suppliers on November 16, 2022; **c.** According to the herein-established optimized protocols.

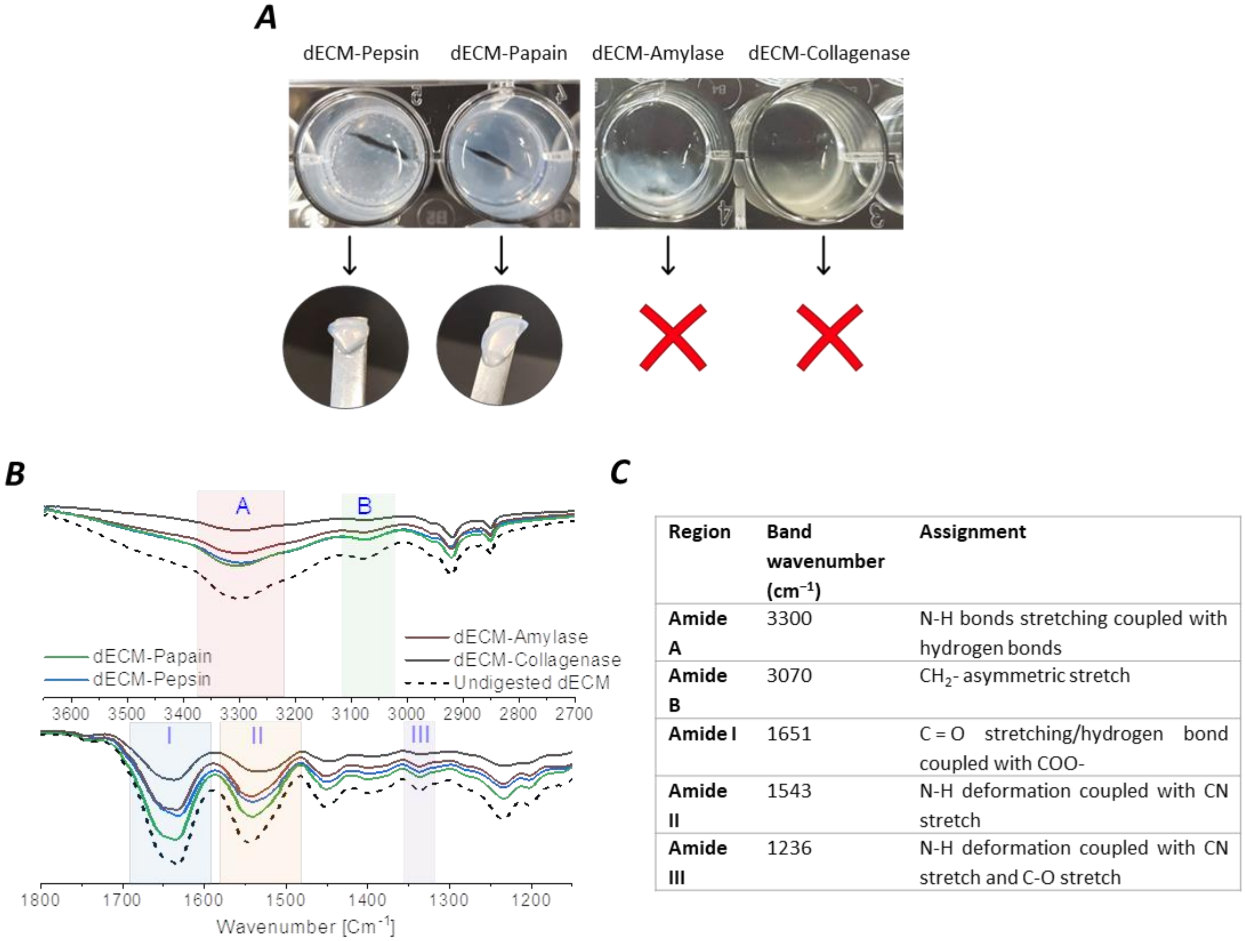

Region	Band wavenumber (cm⁻¹)	Assignment
Amide A	3300	N-H bonds stretching coupled with hydrogen bonds
Amide B	3070	CH_2- asymmetric stretch
Amide I	1651	C = O stretching/hydrogen bond coupled with COO-
Amide II	1543	N-H deformation coupled with CN stretch
Amide III	1236	N-H deformation coupled with CN stretch and C-O stretch

Figure S2. Visual evaluation of the gelation potential of porcine liver dECM digests and FTIR characterization of the dry dECM powder. **(A)** Representative photographs of dECM hydrogels forming at physiological conditions (pH=7.4 and 1x PBS) after 2 h of incubation at 37 °C at a 10 mg/mL pre-gel concentration. **(B)** FTIR spectra of dry dECM-digests showing the presence of collagen characteristic amide bands A and B in the overview spectra shown in the upper panel and the amide bands I, II, and III in the enlargement in the lower panel in comparison to undigested dECM and **(C)** assignments of the characteristic bands.[1-4]

Table S2. Growth factor and cytokine content of porcine native liver tissues compared to dECM samples digested with papain, pepsin, and α-amylase assessed via a multiplex-ELISA-array. A list of growth factor and cytokine full names is provided below the table. Values are means of n=4 ± SD.

Growth Factor	Native ECM [pg/mL]	dECM-Papain [pg/mL]	dECM-Pepsin [pg/mL]	dECM-Amylase	Growth Factor	Native ECM [pg/mL]	dECM-Papain [pg/mL]	dECM-Pepsin [pg/mL]	dECM-Amylase

**AR**	220 ± 36	114 ± 8	17 ± 5 *	213 ± 18	**IGFBP-4**	26507 ± 5241	20124 ± 1903	6323 ± 765	20500 ± 2513
**BDNF**	139 ± 15	110 ± 11	41 ± 7	167 ± 23	**IGFBP-6**	6646 ± 2101	4067 ± 550	1698 ± 357	5627 ± 659
**β-FGF**	138 ± 33	125 ± 16	30 ± 3	157 ± 16	**IGF-I**	553 ± 101	371 ± 84	136 ± 30	437 ± 109
**BMP-4**	397 ± 30	295 ± 51	108 ± 18 *	496 ± 61	**Insulin**	5512 ± 336	2699 ± 87	919 ± 123	3758 ± 123
**BMP-5**	18053 ± 2592	9788 ± 935	3033 ± 538	9699 ± 1063	**MCSF R**	277 ± 25	154 ± 16	63 ± 5	208 ± 24
**BMP-7**	2801 ± 526	2297 ± 164	585 ± 86	1991 ± 368	**NGF R**	231 ± 59	119 ± 10	89 ± 5	132 ± 23
**β-NGF**	43 ± 11	55 ± 5	18 ± 3	75 ± 10	**NT-3**	74 ± 5	42 ± 1	19 ± 2 *	52 ± 8
**EGF**	0.5 ± 0 *	0.4 ± 0 *	0.1 ± 0 *	1 ± 0	**NT-4**	42 ± 3	23 ± 3	11 ± 3	37 ± 3
**EGF R**	182 ± 27	129 ± 20	32 ± 7 *	132 ± 28	**OPG**	15 ± 1	14 ± 3	5 ± 1	19 ± 2
**EG-VEGF**	5 ± 2 *	6 ± 1 *	2 ± 0 *	5 ± 1 *	**PDGF-AA**	1739 ± 210	1036 ± 190	416 ± 36	1677 ± 285
**FGF-4**	126 ± 24	101 ± 13	47 ± 8 *	140 ± 28	**PIGF**	15 ± 3	8 ± 1	3 ± 1	12 ± 1
**FGF-7**	59 ± 11	46 ± 9	15 ± 2	51 ± 3	**SCF**	26 ± 4	22 ± 2	15 ± 1	29 ± 4
**GDF-15**	4 ± 1*	3 ± 0 *	1 ± 0 *	3 ± 0	**SCF R**	256 ± 55	148 ± 11	43 ± 4	263 ± 11
**GDNF**	31 ± 6	21 ± 2	8 ± 1 *	30 ± 5	**TGF-α**	12 ± 1	6 ± 1 *	3 ± 0 *	10 ± 1 *
**GH**	168 ± 31	128 ± 24	58 ± 8	150 ± 12	**TGF-β1**	26025 ± 1830	11787 ± 852	4038 ± 511	13021 ± 427
**HB-EGF**	23 ± 1	16 ± 2	4 ± 1 *	18 ± 2	**TGF-β3**	4251 ± 328	3282 ± 355	1268 ± 226	3397 ± 603
**HGF**	77 ± 10	42 ± 10	19 ± 2	78 ± 11	**VEGF**	14 ± 3	10 ± 2	3 ± 0 *	13 ± 2
**IGFBP-1**	186 ± 9	117 ± 16	51 ± 3	136 ± 23	**VEGF R2**	465 ± 38	392 ± 62	175 ± 22	493 ± 79
**IGFBP-2**	648 ± 110	366 ± 30	95 ± 18	597 ± 85	**VEGF R3**	149 ± 26	144 ± 33	82 ± 12	146 ± 24
**IGFBP-3**	4223 ± 520	3111 ± 432	1406 ± 194	4131 ± 523	**VEGF-D**	26 ± 3	19 ± 5	7 ± 1 *	18 ± 6

* Value is below the detection limit of the kit.

AR, androgen receptor; BDNF, brain-derived neurotrophic factor; BMP, bone morphogenic protein; NGF, neural growth factor; HB, heparin-binding; EGF, epidermal growth factor; FGF, fibroblast growth factor; GDF, growth differentiation factor, GH, growth hormone, OPG, osteoprotegerin; GDNF, glial cell-derived neurotrophic factor; IGFBP, insulin-like growth factor binding protein; IGF, insulin-like growth factor; MCSF, macrophage colony-stimulating factor 1; NT, neurotrophin; PDGF, platelet-derived growth factor; PIGF, placenta growth factor; SCF, stem cell growth factor; TGF, transforming growth factor; VEGF, vascular endothelial growth factor.

Table S3. Importance of growth factors in liver growth and development.[5]

**Growth Factors**	**Name**	**Importance in liver growth and development**

**HGF**	Hepatocyte growth factor	Regulation of cell growth, cell motility, and morphogenesis.
**EGF & EGFR**<br>**HB-EGF**	Epidermal growth factor<br>EGF-receptor<br>Heparin binding-EGF	Stimulation of cell growth, proliferation, and differentiation.
**TGF (α & β)**	Transforming growth factors	Control of cell proliferation, differentiation, adhesion, migration, and embryogenesis.<br>TGFα expression correlates with hepatocyte DNA synthesis during liver development and growth.
**bFGF or FGF**	Basic fibroblast growth factor	FGFs can directly regulate liver fatty acid metabolism and thus prevent hepatic steatosis.
**VEGF-family**	Vascular endothelial growth factors	Regulation of angiogenesis and lymph angiogenesis.
**IGF-family**<br>**and Insulin**	Insulin-like growth factors	Mediate growth-promoting mitogenic effects of growth hormones and are involved in the differentiation and inhibition of apoptosis in various cells; regulation of neural development.

HGF, hepatocyte growth factor; EGF, epidermal growth factor; EGFR, epidermal growth factor receptor; HB-EGF, heparin-binding epidermal growth factor-like growth factor; FGF, fibroblast growth factor; bFGF, basic fibroblast growth factor; IGF, insulin-like growth factor; TGF, transforming growth factor; VEGF, vascular endothelial growth factor.

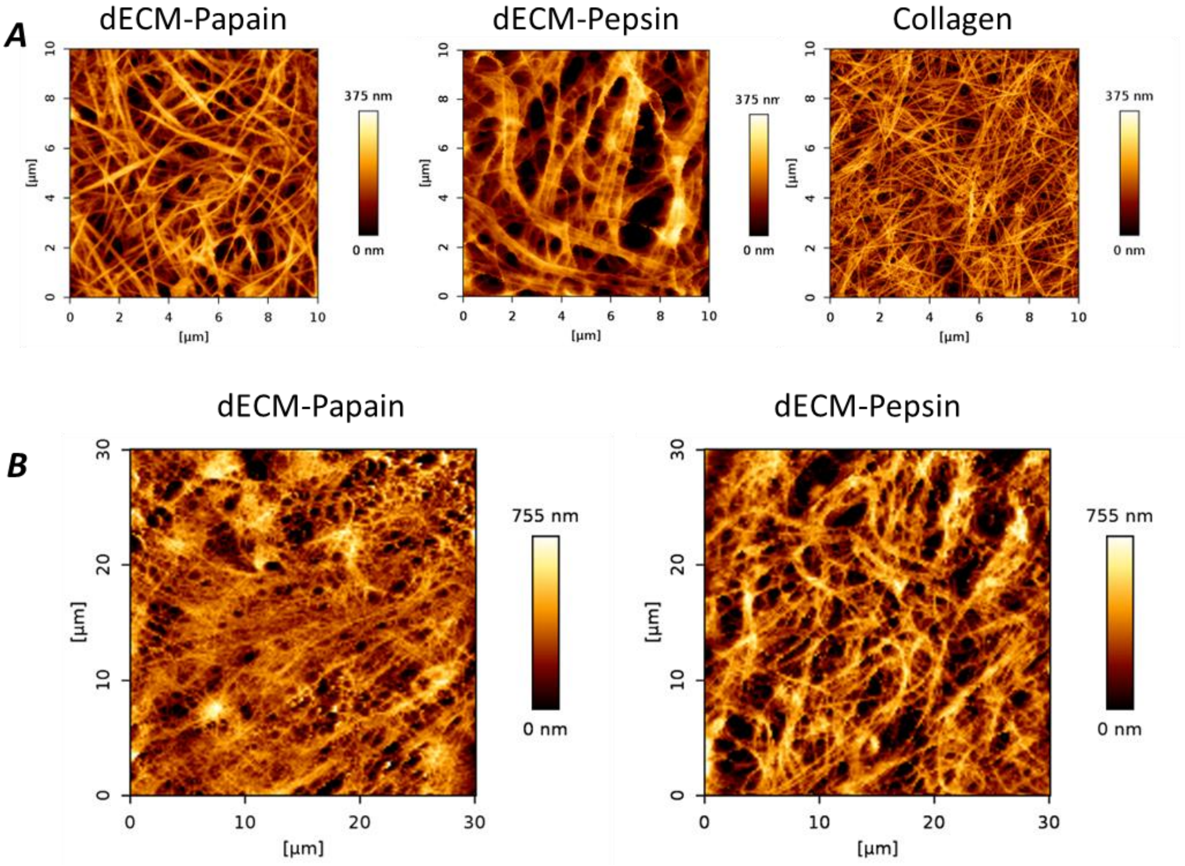

Figure S2. Surface morphology of the wet dECM-based hydrogels (10 mg/mL) with collagen (2.5 mg/mL) as the control accessed via AFM at 37 °C in 1x PBS. **(A)** 10x10 μm and **(B)** 30x30 μm surface area.

References

(1) Chrabaszcz, K.; Kaminska, K.; Augustyniak, K.; Kujdowicz, M.; Smeda, M.; Jasztal, A.; Stojak, M.; Marzec, K. M.; Malek, K. Tracking Extracellular Matrix Remodeling in Lungs Induced by Breast Cancer Metastasis. Fourier Transform Infrared Spectroscopic Studies. *Molecules* **2020**, *25* (1), 236. DOI: 10.3390/molecules25010236.

(2) Cheheltani, R.; McGoverin, C. M.; Rao, J.; Vorp, D. A.; Kiani, M. F.; Pleshko, N. Fourier transform infrared spectroscopy to quantify collagen and elastin in an in vitro model of extracellular matrix degradation in aorta. *Analyst* **2014**, *139* (12), 3039-3047. DOI: 10.1039/c3an02371k.

(3) Habermehl, J.; Skopinska, J.; Boccafoschi, F.; Sionkowska, A.; Kaczmarek, H.; Laroche, G.; Mantovani, D. Preparation of ready-to-use, stockable and reconstituted collagen. *Macromol Biosci* **2005**, *5* (9), 821-828. DOI: 10.1002/mabi.200500102.

(4) Ahn, G.; Min, K. H.; Kim, C.; Lee, J. S.; Kang, D.; Won, J. Y.; Cho, D. W.; Kim, J. Y.; Jin, S.; Yun, W. S.; et al. Precise stacking of decellularized extracellular matrix based 3D cell-laden constructs by a 3D cell printing system equipped with heating modules. *Sci Rep* **2017**, *7* (1), 8624. DOI: 10.1038/s41598-017-09201-5.

(5) Hoffmann, K.; Nagel, A. J.; Tanabe, K.; Fuchs, J.; Dehlke, K.; Ghamarnejad, O.; Lemekhova, A.; Mehrabi, A. Markers of liver regeneration-the role of growth factors and cytokines: a systematic review. *BMC Surg* **2020**, *20* (1), 31. DOI: 10.1186/s12893-019-0664-8.
